# Integrating Genome-Wide Association Studies and Gene Expression Profiles With Chemical-Genes Interaction Networks to Identify Chemicals Associated With Colorectal Cancer

**DOI:** 10.3389/fgene.2020.00385

**Published:** 2020-04-24

**Authors:** Xinyue Tan, Hanmin Tang, Liuyun Gong, Lina Xie, Yutiantian Lei, Zhenzhen Luo, Chenchen He, Jinlu Ma, Suxia Han

**Affiliations:** Department of Oncology, The First Affiliated Hospital, Xi’an Jiaotong University, Xi’an, China

**Keywords:** colorectal cancer, genome-wide association study, transcriptome-wide association study, gene set enrichment analysis, comparative toxicogenomics database

## Abstract

Colorectal cancer (CRC) is the third most common cancer and has the second highest mortality rate in global cancer. Exploring the associations between chemicals and CRC has great significance in prophylaxis and therapy of tumor diseases. This study aims to explore the relationships between CRC and environmental chemicals on genetic basis by bioinformatics analysis. The genome-wide association study (GWAS) datasets for CRC were obtained from the UK Biobank. The GWAS data for colon cancer (category C18) includes 2,581 individuals and 449,683 controls, while that of rectal cancer (category C20) includes 1,244 individuals and 451,020 controls. In addition, we derived CRC gene expression datasets from the NCBI-GEO (GSE106582). The chemicals related gene sets were acquired from the comparative toxicogenomics database (CTD). Transcriptome-wide association study (TWAS) analysis was applied to CRC GWAS summary data and calculated the expression association testing statistics by FUSION software. We performed chemicals related gene set enrichment analysis (GSEA) by integrating GWAS summary data, mRNA expression profiles of CRC and the CTD chemical-gene interaction networks to identify relationships between chemicals and genes of CRC. We observed several significant correlations between chemicals and CRC. Meanwhile, we also detected 5 common chemicals between colon and rectal cancer, including methylnitronitrosoguanidine, isoniazid, PD 0325901, sulindac sulfide, and importazole. Our study performed TWAS and GSEA analysis, linked prior knowledge to newly generated data and thereby helped identifying chemicals related to tumor genes, which provides new clues for revealing the associations between environmental chemicals and cancer.

## Introduction

Colorectal cancer (CRC) is the third most common cancer worldwide and has the second highest mortality rate in global cancer (Bray et al., 2018; Ferlay et al., 2019). In western countries, CRC accounts for about 10% of cancer deaths (Kuipers et al., 2015). The accepted view is that genetic, lifestyle, and environmental factors are closely related to CRC (Dekker et al., 2019). Current research shows that environmental chemicals play important roles in the etiology of CRC. Several chemicals have been suggested to promote the tumorigenesis and development of CRC. For instance, analysis of an Iowa Women’s Health Study cohort suggested that exposure to TTHM in drinking water is associated with increased risk of rectal cancer (Jones et al., 2019). In addition, another case-control study observed that organochlorine and organophosphorus pesticides may induce CRC (Abolhassani et al., 2019). In contrast, numerous chemicals have been identified that inhibit CRC. Metastatic CRC (mCRC) often indicates a poor prognosis. The 5-year overall survival (OS) rate of patients with mCRC is less than 15% (Siegel et al., 2017; Bray et al., 2018), and the median OS of unresectable mCRC patients who received only supportive therapy was only 5 months (Lucas et al., 2011). However, the 5-years OS rate increased to 10% in such patients receiving 5-fluorouracil (5-FU)/leucovorin (LV) plus oxaliplatin (FOLFOX) (Gustavsson et al., 2015). Thus, FOLFOX chemotherapy regimen is still the standard first-line therapy for unresectable mCRC (Giacchetti et al., 2000; Goldberg et al., 2004; Kouhara et al., 2007; Bokemeyer et al., 2011). Recent studies have found that some non-chemotherapeutic chemicals also have an inhibitory effect on CRC, such as semisynthetic retinoid, lidocaine, and beta-carotene (Mattingly et al., 2003; Pham et al., 2013; Qu et al., 2018).

Therefore, it has great significance to clarify the relationship between chemicals in environmental and CRC for the treatment and prevention of diseases. But obtaining the entire life-time exposure of an individual is difficult and complex, for lacking sensitive methods to measure specific exposures. Although the exposure is known to have occurred, the transient character of the exposure indicators increases the difficulty of measuring the specific exposure (Messerlian et al., 2017). With the help of new technologies, such as genome-wide association research (GWAS), more convenient and efficient analyses have been produced to identify interactions between multiple environmental exposures with genes (Khoury et al., 2005). Studies of gene-environment interactions have been widely applied in psychological research, however, lack in the field of oncology (Manuck and McCaffery, 2014). The Comparative Toxicogenomics Database (CTD) is a public repository, aims to advance people’s understanding of how environmental exposures affects human health (Mattingly et al., 2003). This database provides information regarding chemical-gene/protein interactions as well as chemical- and gene-disease relationships that is organized by individual genes, gene sets, organisms, chemicals, sequence type (DNA, mRNA, and protein), gene ontology annotations and sequences (Mattingly et al., 2006).

Genome-wide association studies (GWAS) analyze DNA sequence variations to provide associations for complex human traits and diseases efficiently (Tam et al., 2019). Transcriptome-wide association studies (TWAS) is further developed on this basis, which can evaluate the association of each gene to diseases by integrating tissue-related gene expression measurements with GWAS summary data (Gong et al., 2018). Currently, TWAS has been proved with high efficiency in determining the genetic mechanism of complex diseases (Gusev et al., 2018; Wu et al., 2018). The Gene Expression Omnibus (GEO) is a worldwide resource which distributes a large number of high-throughput microarray and next-generation sequence functional genomic data sets (Barrett et al., 2013). Different from the traditional GWAS to explain the relationship between DNA and external phenotype, we simultaneously used the GEO to obtain the gene expression profile (mRNA expression profile chip data) of colorectal cancer, that is, a comprehensive analysis at the DNA and mRNA level. This is helpful to narrow the range of chemicals related candidate genes on the basis of traditional GWAS analysis.

Briefly, in this work, the CTD chemical-gene interaction networks, GWAS summary datasets and gene expression profiles were integrated. TWAS analysis was performed by FUSION software to evaluate the expression association testing statistics. The gene set enrichment analysis (GSEA) with the running-sum statistic and weighted Kolmogorov-Smirnov–like statistic were applied to detect the correlation between environmental chemicals and CRC (Charmpi and Ycart, 2015). Firstly, we obtained the empirical distributions of GSEA statistics for each chemical for statistical tests. Subsequently, the *P-*value of each chemical was conducted from the permuted empirical distribution of GSEA statistics. Finally, we summarized and analyzed the obtained chemicals associated with CRC.

## Materials and Methods

### GWAS Summary Dataset for CRC

GeneATLAS^1^, a huge resource storing the information of hundreds of traits and millions of related gene variants based on the UK Biobank cohort, provides a convenient way for researchers to acquire data from the UK Biobank (Lin et al., 2019). To be specific, it allows researchers to query genome-wide association results for 9,113,133 genetic variants and download over 30 million genetic variants (>23 billion phenotype-genotype pairs) for GWAS summary statistics (Canela-Xandri et al., 2018).

A large-scale GWAS summary data of colon cancer and rectal cancer in our study were downloaded from the GeneATLAS in UK Biobank. In the cancer register category, 103,470 data items are available from 84,726 participants. In brief, our GWAS summary data, which contained 5,899 available data items, were from categories C18 (malignant neoplasm of colon) and C20 (malignant neoplasm of rectum). Detailed information regarding the methods, process, and approaches were described in the previous studies (Hammerschlag et al., 2017).

### Gene Expression Datasets of CRC

NCBI-GEO^2^ is an international public repository with next-generation sequencing and microarray/gene profiles which was used in this study to obtain the mRNA expression profiles of mucosa and colorectal tumor tissues (GSE106582). CRC patients were recruited at the University Hospital of Heidelberg, from whom the gene expression profiles of 77 tumor and 117 mucosa tissues were obtained using an Illumina HumanHT-12 V4.0 expression beadchip. Using GEO2R, a web tool based on the GEO database, differential gene expression was assessed by comparing the expression of genes from colorectal tumor tissues to those of respective mucosa tissues.

### Transcriptome-Wide Association Study (TWAS) Methodology

TWAS analysis utilizes disease GWAS summary statistics combining with pre-computed gene expression weights to calculate the association of every gene with known diseases (Gusev et al., 2018). In other words, TWAS can integrate the associations between GWAS and gene expression measurements to identify genes associated with traits. In this study, A TWAS for CRC was conducted using functional summary-based imputation (FUSION) software and the gene expression weight references of whole blood, rectum, and colon tissues were acquired from the FUSION website^3^. Specifically, the gene expression weights of whole blood were collected from 1,264 subjects of the Young Finns Study (Raitakari et al., 2008; Nuotio et al., 2014).

Firstly, based on FUSION software we performed prediction models to calculate the gene expression weights of different tissues (Huang et al., 2019). Then we conducted the correlation statistics between gene expressed level and CRC combining the gene expression weights and summary-level GWAS results. Z_*TWAS*_ = w’Z/(w’Lw)^1/2^ was used to calculate the association statistics. Z denotes the scores of CRC while w denotes the weights. L means the SNP-correlation linkage disequilibrium (LD) matrix. A TWAS *p-*value was calculated for each gene within whole blood, rectum and colon tissues, respectively (Qi et al., 2019). The genes with *p* < 0.05 were considered as significant. Detailed information can be found in the published study (Gusev et al., 2018).

### Chemical-Gene Expression Interaction Database of the CTD Database

The Comparative Toxicogenomics Database (CTD)^4^ is a publicly and accessible database for toxicogenomic information (Zhang et al., 2019). The CTD currently includes more than 30.5 million toxicogenomic relationships associated with chemicals, proteins, etc. (Davis et al., 2017) and provides information regarding chemical, gene, phenotype, and disease relationships to advance our understanding of the effects of environmental toxin exposure on public health (Grondin et al., 2018). A unique and powerful feature of the CTD is knowledge transfer with respect to any information that is directly annotated to chemicals, genes and diseases (Davis et al., 2013). This study download 11,190 chemicals related gene sets from the CTD. The process of retrieving information using CTD was described in the study previously (Mattingly et al., 2006).

### Identification of Environmental Chemicals Elements Associated With Colorectal Cancer

The GSEA algorithm was originally used for microarray study and GWAS-based GSEA was developed subsequently (He et al., 2018). At present, it is utilized to identify abnormally expressed gene sets for target diseases, and has been applied in etiology researches of multiple diseases (Wang et al., 2007). Firstly, for the *j*th (j = 1,2,3…*N*) gene, the most significant GWAS association test statistics of the SNPs was assigned to *j*th gene according to the score r_j_ of the given gene. Secondly, all genes G = (G_1^*_, G_2*_,…,G_N^*_) were ranked by their scores from the highest to the lowest (He et al., 2018), which was expressed as U = (j_1^*_, j_2^*_,…,j_N^*_). Thirdly, for a chemicals related gene set *S*, an enrichment score *ES* was calculated for CRC by the running sum statistic and weighted Kolmogorov-Smirnov-like statistic (Subramanian et al., 2005; Charmpi and Ycart, 2015). Gene set S independently derived from *N*_*H*_ genes. *ES* represents the overrepresentation of CRC associated genes in chemicals related gene set S. *ES* was calculated as:

$$\text{ES}\,\left(S\right)=\max_{1\leq j\leq N}\left\{\sum_{G_{j^{*}}\in S,j^{*}\leq j}\frac{{\left|{\text{r}}_{j^{*}}\right|}^{p}}{{\text{N}}_{R}}-\sum_{G_{j^{*}}\notin S,j^{*}\leq j}\frac{1}{\text{N}-{\text{N}}_{H}}\right\},$$

where

$${\text{N}}_{R}=\sum_{G_{j}\in S}{\left|{\text{r}}_{j^{*}}\right|}^{p}.$$

Finally, after L time permutations, we can obtain the null distribution of $E\,S^{n\,u\,l\,l}=\left(E\,S_{1}^{n\,u\,l\,l},E\,S_{2}^{n\,u\,l\,l},\ldots ,E\,S_{l}^{n\,u\,l\,l}\right)$. To control the effect of the gene sets with varying sizes, the observed *ES*(*S*) is normalized by the average value and standard deviation of the permutated $E\,S_{S}^{n\,u\,l\,l}$, defined by $N\,E\,S^{S}=\frac{E\,S^{S}-m\,e\,a\,n\,\left(E\,S_{S}^{n\,u\,l\,l}\right)}{S\,D\,\left(E\,S_{S}^{n\,u\,l\,l}\right)}$. The *P-*values were finally calculated from the NES for each chemicals related gene set.

This study conducted a total of 5,000 permutations to calculate the empirical distributions of GSEA statistics of each chemical. And the chemicals related gene sets with *P* < 0.05 are considered statistically significant. Previous research provides the detailed descriptions regarding this approach (Zhao et al., 2018). Similarly, all mRNA expression profile from GEO were analyzed using the same approach (Weng et al., 2011).

## Results

### Environmental Chemicals Associated With Colorectal Cancer

From the CRC GWAS summary datasets, we identified 175 chemicals that were significantly associated with colon cancer (including 34 for colon tissue and 141 for whole blood) as well as 103 chemicals significantly associated with rectal cancer (including 20 for rectal tissue and 83 for whole blood) (*P* < 0.05; Supplementary Tables S1, S2). For the expression profile of CRC, we identified 1,198 significant chemicals (*P* < 0.05; Supplementary Table S3).

After a comparative analysis of the TWAS and mRNA expression profile GSEA results, we significantly detected several chemicals associated with the colon cancer and rectal cancer (*P* < 0.05). For colon cancer, 104 common chemicals were detected, including 83 in colon tissue and 24 in blood tissue, and 3 in both tissues (Supplementary Table S4),such as Antirheumatic Agents (*P*-value1 = 0.0244, *P-*value2 = 0.0230), Chenodeoxycholic Acid (*P-*value1 = 0.0002, *P-*value2 = 0.0002) and Trientine (*P-*value1 = 0.0464, *P-*value2 = 0.0314; Supplementary note: In this paragraph, *P-*value1 is *P-*value in GWAS dataset and *P-*value2 is *P-*value in mRNA expression profile). For rectal cancer, 51 common chemicals were discovered, including 12 in rectum tissue and 39 in blood tissue (Supplementary Table S5). Tables 1, 2 summarized the top 10 chemicals identified for the colon cancer and rectal cancer separately.

**TABLE 1:** List of top ten chemicals identified for colon cancer after a comparative of GWAS and mRNA GSEA results.

Chemical Name	*P*-value1^a^	*P*-value2^b^
Antirheumatic Agents	0.0002	0.0002
LG 100815	0.0004	0.0004
Zinc Acetate	0.0010	0.0004
Aerosols	0.0016	0.0002
Titanium dioxide	0.0026	0.0002
Motexafin gadolinium	0.0046	0.0006
Clofibric Acid	0.0052	0.0002
Vitallium	0.0052	0.0002
Raloxifene Hydrochloride	0.0066	0.0002
Soman	0.0094	0.0002

*^*a*^P-value1: P-value in GWAS dataset. ^*b*^P-value2: P-value in mRNA expression profile.*

**TABLE 2:** List of top ten chemicals identified for rectal cancer after a comparative of GWAS and mRNA GSEA results.

Chemical name	*P-*value1^a^	*P*-value2^b^
NAD	0.0020	0.0004
Sulindac sulfide	0.0052	0.0002
Casticin	0.0086	0.0002
Benz(a)anthracene	0.0124	0.0002
Methylnitronitrosoguanidine	0.0132	0.0002
Afimoxifene	0.0134	0.0002
4-phenylbutyric acid	0.0150	0.0004
Nickel	0.0178	0.0002
Ochratoxin A	0.0180	0.0002
Promethazine	0.0196	0.0006

*^*a*^P-value1: P-value in GWAS dataset. ^*b*^P-value2: P-value in mRNA expression profile.*

**FIGURE 1:**
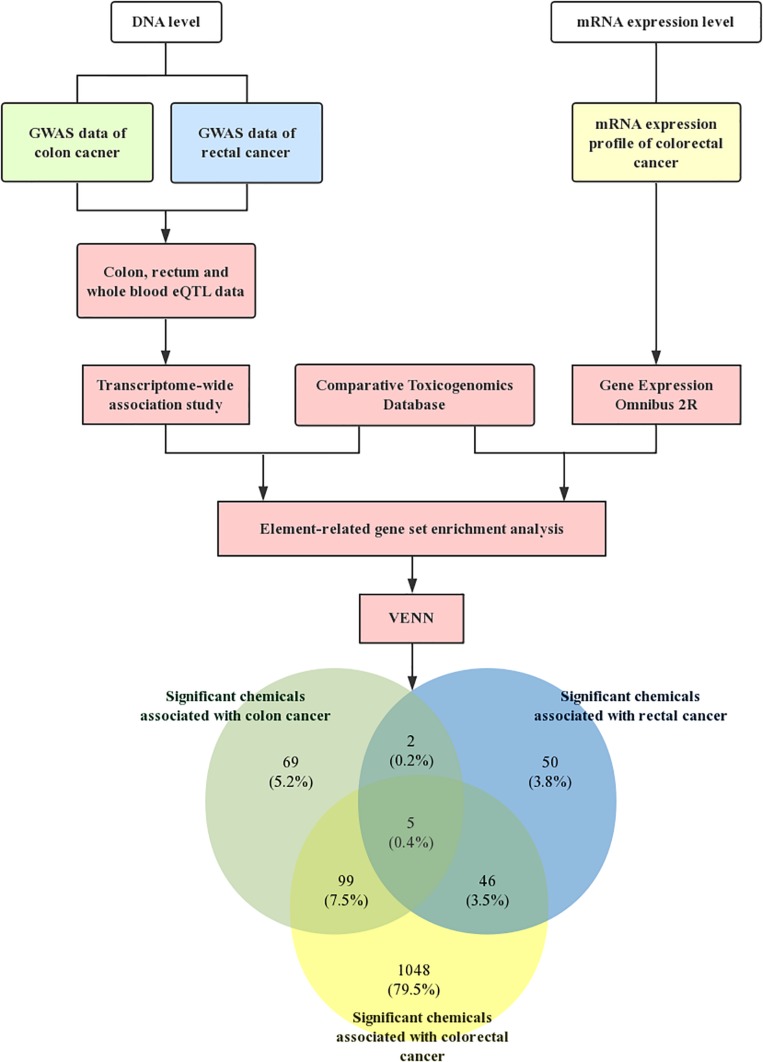
Technology roadmap. First, the GWAS dataset of colon cancer and rectal cancer were downloaded from GeneATLAS, a large database based on the UK Biobank cohort. Meanwhile, we obtained mRNA expression profiles of CRC from NCBI-GEO. The software FUSION was used to assess the CRC GWAS summary data for tissue-related TWAS analysis. The chemicals related gene sets were then generated by the CTD. Subsequently, chemical-related gene set enrichment analysis (GSEA) was conducted to detect the association between chemicals and CRC. Finally, the Venn diagram showed the significant chemicals associated with colorectal cancer.

Meanwhile, Table 3 shows the common significant environmental chemicals between colon cancer and rectal cancer. We detected 5 chemicals, including methylnitronitrosoguanidine (*P-*value1 = 0.0394, *P-*value2 = 0.0132, *P-*value3 = 0.0002), isoniazid (*P-*value1 = 0.0164, *P-*value2 = 0.0262, *P-*value3 = 0.0068), PD 0325901 (*P-*value1 = 0.0348, *P-*value2 = 0.0406, *P-*value3 = 0.0012), sulindac sulfide (*P-*value1 = 0.0374, *P-*value2 = 0.0052, *P-*value2 = 0.0002), importazole (*P-*value1 = 0.0378, *P-*value2 = 0.0450, *P-*value3 = 0.0224; Supplementary note: In this paragraph, *P-*value1 is *P-*value in colon GWAS dataset, *P-*value2 is *P-*value in rectal GWAS dataset, *P-*value3 is *P-*value in mRNA expression profile). The specific technology roadmap and Venn diagram are shown in Figure 1.

**TABLE 3:** The common significant chemicals between colon cancer and rectal cancer GSEA results.

Chemical name	*P*-value1^a^	*P*-value2^b^	*P*-value3^c^
Methylnitronitrosoguanidine	0.0394	0.0132	0.0002
Isoniazid	0.0164	0.0262	0.0068
PD 0325901	0.0348	0.0406	0.0012
Sulindac sulfide	0.0374	0.0052	0.0002
Importazole	0.0378	0.0450	0.0224

*^*a*^P-value1: P-value in colon GWAS dataset. ^*b*^P-value2: P-value in rectal GWAS dataset. ^*c*^P-value3: P-value in mRNA expression profile.*

## Discussion

CRC is the fourth deadliest cancer lead to 900,000 deaths worldwide annually (Dekker et al., 2019). It has become a global public health problem due to its high morbidity and mortality worldwide. Both genetic and environmental factors play significant roles in the etiology of colorectal cancer. Cancer risk factors include biological agents (infection), exposure to synthetic chemicals, and lifestyle factors, which together contribute to the development of 70–95% of cancers (Wu et al., 2016). Several chemicals have been reported promote the tumorigenesis and tumor development of CRC (Abolhassani et al., 2019; Cernigliaro et al., 2019; Jones et al., 2019). This provides a new clue for us to prevent the occurrence of colorectal cancer. Meanwhile, except for the standard treatment, many chemicals have been reported to inhibit CRC in recent years. For example, the anti-colorectal cancer effect of awsonaringenin (LSG), a flavonoid compound, has been demonstrated in previous research (Anwar et al., 2018). Environmental chemicals are related to various malignant tumors besides CRC. For example, acrylamide, benzo(a)pyrene and polychlorinated biphenyls can induce carcinogenesis for cytotoxicity and DNA damage to hepatic cells (Erkekoglu et al., 2017). The discovery for the active substance in chemicals related cancer is of great significance for the treatment to tumor patients. Since the chemicals environmental exposure is usually complex and accurately measuring exposure levels *in vivo* is still with many objective problems, we try to explore the relationships between chemicals and cancer in an easier way.

In this study, we extended the classical GSEA approach to detect associations between chemicals and CRC using TWAS data and gene expression datasets. We identified several chemicals showing genetic correlation evidence with the CRC.

We identified several significant chemicals for the colon cancer, such as aspirin and titanium dioxide, which have been reported by previous study. Aspirin, a well-known antirheumatic drug, is proved that can prolong the survival of patients with colorectal cancer and activate T cell-mediated antitumor immunity (Hamada et al., 2017). Bettini, Boutet-Robinet et al. has reported that daily oral food-grade titanium dioxide (TiO2) intake is related to an chronic intestinal inflammation and will increase the risk of carcinogenesis (Bettini et al., 2017).

NAD and Nickel are two remarkable chemicals associated with rectal cancer. A recent study revealed that increased nicotinamide adenine dinucleotide pool suppressed reactive oxygen species level to promote progression of colon cancer (Hong et al., 2019). In a previous study, trace elements in normal and cancerous tissue which obtained from 18 patients suffering from colon and rectum cancer were quantitatively determined by X-ray fluorescence, and the result showed that Nickel elevated in cancerous tissues (Gregoriadis et al., 1983).

Five overlapped chemicals have been identified associated with CRC, including the carcinogens methylnitronitrosoguanidine, isoniazid. And PD 0325901, sulindac sulfide and importazole have the ability to inhibit the carcinogenesis and development of cancer.

Methylnitronitrosoguanidine (MNNG) is anticipated to be declared a human carcinogen based on sufficient evidence of its carcinogenicity from investigations involving animal models. MNNG caused tumors at different tissue sites in several animal model species by several different exposure routes. Research indicated that the intrarectal infusion of MNNG into large intestine of rats can cause tumors (Tsukamoto et al., 2015; U.S. Department of Health and Human Services, 2016).

Isoniazid (INH) is an irreversible inhibitor of Monoamine oxidase A (MAOA) that is widely regarded as a major anti-tuberculosis drug (Zareifopoulos and Panayiotakopoulos, 2017). MAOA is a mitochondrial-bound enzyme. It was confirmed that MAOA may promote the progression of prostate cancer by mediating EMT (Wu et al., 2014; Lv et al., 2018). However, because conflicting results have been reported for the importance of MAOA in HCC and cholangiocarcinoma (Huang et al., 2012; Li et al., 2014), the role of MAOA may vary across cancer types. Lee et al. demonstrated that Monoamine Oxidase Inhibitors (MAOIs) are associated with increased colorectal cancer risk (adjusted OR = 1.22, 95% CI = 1.06-1.41; Lee et al., 2017).

PD 0325901 is an MEK inhibitor. Interestingly, Roper et al. (2014) have shown PI3K/MEK inhibition combined with NVP-BKM120 and PD-0325901 treatment can induce tumor progression in a wild-type PIK3CA mouse model, KRAS mutant CRC, based on the inhibition of mTORC1 and MCL-1 and the activation of BIM. Moreover, PD0325901 was reported to inhibit oxaliplatin-induced neuropathy and enhance oxaliplatin efficacy (Tsubaki et al., 2015).

Liggett et al. observed that the non-steroidal anti-inflammatory drug sulindac sulfide inhibits the expression of the potential oncogene structural protein nesprin-2 in CRC cells (Liggett et al., 2014). The results of another study suggested the inhibition of sulindac sulfide on the growth of colon cancer cells and down-regulation of specific transcription factors (Li et al., 2015). Furthermore, the inhibitory effects of 5-fluorouracil and oxaliplatin on human CRC cell survival were demonstrated to be synergistically enhanced by sulindac sulfide (Flis and Splwinski, 2009).

Importazole is a small molecule inhibitor of the transport receptor importin-β (Soderholm et al., 2011) that can inhibit the proliferation and induce apoptosis of multiple myeloma cells by blocking the NF-KB signaling pathway (Yan et al., 2015). Moreover, intravenous administration of the specific KPNB1 inhibitor importazole was effective in reducing the volume and weight of prostate cancer tumor in mice inoculated with PC3 PCa cells (Yang et al., 2019). Thus, the results of the above studies show that importazole can inhibit tumors.

We conducted a large scale correlation study between colorectal cancer and environmental chemicals and explored the associations between chemicals and colorectal cancer systematically. Our analysis approach has two advantages. Firstly, we identified interaction between chemicals and genes directly. From the perspective of genome, the result is more stable to overcome the shortcomings of traditional exposure measurement methods. From the perspective of benefit, genome-wide summary data usually can be obtained online conveniently. Secondly, our research analyzed summaries of TWAS and mRNA expression profiles, in other words, we made a comprehensive analysis in the DNA and mRNA expression levels. This is helpful to narrow the range of chemicals related candidate genes on the basis of traditional GWAS analysis and make the results more reliable. Current research shows that chemicals in environmental factors have great significance in the etiology of multiple cancers (Thompson et al., 2015). However, we only researched the colon cancer and rectal cancer. As cancer sequencing gene data sets increasing, we will apply our method to large-scale studies of cancer gene-environment interactions.

In summary, we conducted an integrative analysis of GWAS summary data, mRNA expression profiles and chemical-gene interaction networks. Tools such as TWAS and GSEA helped linking these datasets and identifying several chemicals associated with CRC. The results of our study evaluate the associations between CRC and chemicals systematically, and provide new clues for revealing the association between chemicals and genes and their effects on cancer. Furthermore, our method can be used to analyze other chemicals and complex malignant disease, which is helpful for assessing the relationship between environmental exposure and cancer.

## Data Availability Statement

Publicly available datasets were analyzed in this study. This data can be found here: https://www.ncbi.nlm.nih.gov/gds/ (GSE106582), http://geneatlas.roslin.ed.ac.uk/ (categories C18, categories C20), http://gusevlab.org/projects/fusion/, http://ctdbase.org/.

## Author Contributions

XT and SH designed experiments. XT, HT, ZL, and YL reviewed and downloaded the original data. XT, LG, and LX processed and analyzed the data. XT, CH, and JM analyzed experimental results. XT, HT, and SH wrote the manuscript.

## Conflict of Interest

The authors declare that the research was conducted in the absence of any commercial or financial relationships that could be construed as a potential conflict of interest.

## Footnotes

1 http://geneatlas.roslin.ed.ac.uk/

2 https://www.ncbi.nlm.nih.gov/gds/

3 http://gusevlab.org/projects/fusion/

4 http://ctdbase.org/
