# Supplementary material for: Integrating Genome-Wide Association Studies and Gene Expression Profiles With Chemical-Genes Interaction Networks to Identify Chemicals Associated With Colorectal Cancer
Source: Front Genet. 2020 Apr 24;11:385. doi: 10.3389/fgene.2020.00385 (PMC7193025; doi:10.3389/fgene.2020.00385)
Supplement: Supplementary file 1 [file Data_Sheet_1.docx]

Supplementary Material

# Supplementary Tables

## Supplementary Table 1. 175 significant chemicals related to GWAS summary datasets of colon cancer. (P value＜0.05)

| Chemical Name | Chemical ID^1^ | NES^2^ | P value |
| --- | --- | --- | --- |
| Antirheumatic Agents | D018501 | 3.580204796 | 0.00019996 |
| LG 100815 | C533894 | 3.213178855 | 0.00039992 |
| Ethacrynic Acid | D004976 | 3.222770607 | 0.00039992 |
| salinomycin | C010327 | 3.251801298 | 0.00079984 |
| selenomethylselenocysteine | C002979 | 2.945912524 | 0.0009998 |
| polydatin | C058229 | 3.066054217 | 0.0009998 |
| Zinc Acetate | D019345 | 2.95392756 | 0.0009998 |
| Aerosols | D000336 | 3.125169226 | 0.00159968 |
| Am 580 | C068073 | 2.870483929 | 0.00239952 |
| titanium dioxide | C009495 | 2.909921253 | 0.00259948 |
| Arachidonic Acid | D016718 | 2.705408412 | 0.00319936 |
| Calcimycin | D000001 | 2.743250469 | 0.00339932 |
| acteoside | C058956 | 2.529640347 | 0.00359928 |
| butylidenephthalide | C026105 | 2.573603218 | 0.00419916 |
| motexafin gadolinium | C437683 | 2.549601898 | 0.00459908 |
| gallocatechol | C057580 | 2.429807733 | 0.00519896 |
| Catechin | D002392 | 2.614561613 | 0.00519896 |
| Clofibric Acid | D002995 | 2.603845509 | 0.00519896 |
| Carboplatin | D016190 | 2.572215241 | 0.00519896 |
| Pentylenetetrazole | D010433 | 2.515404507 | 0.00619876 |
| Carcinogens | D002273 | 2.420210743 | 0.00659868 |
| Raloxifene Hydrochloride | D020849 | 2.607167242 | 0.00659868 |
| Methylene Blue | D008751 | 2.288763467 | 0.00679864 |
| Omeprazole | D009853 | 2.493032136 | 0.0069986 |
| Cotinine | D003367 | 2.475906652 | 0.00759848 |
| Orotic Acid | D009963 | 2.235435114 | 0.00759848 |
| Cysteine | D003545 | 2.359024303 | 0.00779844 |
| triacsin C | C034613 | 2.363788194 | 0.0089982 |
| icariin | C056599 | 2.230150992 | 0.00919816 |
| aristolochic acid II | C042310 | 2.310887364 | 0.00939812 |
| Oxaliplatin | D000077150 | 2.28101611 | 0.01179764 |
| Triterpenes | D014315 | 2.218425381 | 0.01179764 |
| Cyclophosphamide | D003520 | 2.341800365 | 0.01219756 |
| U 0126 | C113580 | 2.252461116 | 0.012797441 |
| Tretinoin | D014212 | 2.189400937 | 0.013797241 |
| 1-methylanthracene | C051246 | 2.21576877 | 0.014797041 |
| SCH772984 | C587178 | 2.106292339 | 0.015196961 |
| Acrolein | D000171 | 2.160299408 | 0.015196961 |
| Methylmethacrylate | D020366 | 2.092497223 | 0.015796841 |
| ferric nitrilotriacetate | C020326 | 2.149033392 | 0.015996801 |
| Isoniazid | D007538 | 2.147577171 | 0.016396721 |
| aniline | C023650 | 2.164803907 | 0.016596681 |
| Graphite | D006108 | 2.13149168 | 0.016996601 |
| tetradecabromodiphenoxybenzene | C582340 | 2.029570093 | 0.017396521 |
| 2-(4-((4-(6-methoxy-3-pyridinyl)-5-(4-(trifluoromethoxy)phenyl)-2-thiazolyl)methoxy)-2-methylphenoxy)acetic acid | C573693 | 2.056589474 | 0.018396321 |
| Ethionine | D005001 | 2.119504272 | 0.018396321 |
| bromodichloromethane | C025191 | 2.023108743 | 0.018596281 |
| Gliotoxin | D005912 | 2.022635641 | 0.018596281 |
| archazolid B | C519728 | 2.002002784 | 0.019396121 |
| trimethyltin | C046488 | 2.061838249 | 0.019796041 |
| taxifolin | C003377 | 1.991294455 | 0.019996001 |
| Mercuric Chloride | D008627 | 2.024498934 | 0.020595881 |
| Topotecan | D019772 | 2.059987744 | 0.020995801 |
| nickel monoxide | C028007 | 2.028730322 | 0.022195561 |
| lactacystin | C067713 | 2.027308695 | 0.022195561 |
| Chloroprene | D002737 | 2.015718994 | 0.022395521 |
| CP-654577 | C477330 | 1.923234672 | 0.022795441 |
| Vigabatrin | D020888 | 2.026589986 | 0.022795441 |
| systhane | C446685 | 1.967991472 | 0.023195361 |
| bis(4-hydroxyphenyl)sulfone | C543008 | 2.038313193 | 0.023195361 |
| 1-nitropyrene | C032668 | 1.935218119 | 0.023395321 |
| neoechinulin A | C490266 | 1.951861225 | 0.023395321 |
| Bromine | D001966 | 1.934406165 | 0.023795241 |
| Pentoxifylline | D010431 | 1.953404358 | 0.023795241 |
| Letrozole | D000077289 | 1.918194888 | 0.024395121 |
| Chenodeoxycholic Acid | D002635 | 1.976479209 | 0.024395121 |
| Fungal Polysaccharides | D062610 | 1.94218279 | 0.024395121 |
| salubrinal | C496827 | 1.958242278 | 0.024995001 |
| gold (III) porphyrin 1a | C508665 | 1.922389236 | 0.024995001 |
| Lidocaine | D008012 | 2.003732115 | 0.025794841 |
| Diamide | D003958 | 1.938230059 | 0.025994801 |
| Imatinib Mesylate | D000068877 | 1.93358209 | 0.026794641 |
| thymoquinone | C003466 | 1.926973071 | 0.026994601 |
| 4-biphenylamine | C006757 | 1.896433749 | 0.026994601 |
| 4-cresol | C032538 | 1.882883294 | 0.027594481 |
| S-nitro-N-acetylpenicillamine | C110881 | 1.923218162 | 0.027994401 |
| monomethyl phthalate | C517284 | 1.950209504 | 0.028194361 |
| Benzbromarone | D001553 | 1.936041146 | 0.028594281 |
| Minocycline | D008911 | 1.887354558 | 0.028794241 |
| Ivermectin | D007559 | 1.947363403 | 0.029194161 |
| Pilocarpine | D010862 | 1.857844633 | 0.029194161 |
| Benzo(a)pyrene | D001564 | 1.892563854 | 0.029394121 |
| AZM551248 | C547126 | 1.881933773 | 0.029794041 |
| Citrinin | D002953 | 1.850089305 | 0.030993801 |
| Methyltestosterone | D008777 | 1.872855611 | 0.031393721 |
| Selenium | D012643 | 1.874369612 | 0.031393721 |
| Povidone | D011205 | 1.85360318 | 0.032193561 |
| Deoxycholic Acid | D003840 | 1.886804977 | 0.032593481 |
| fludarabine | C024352 | 1.836658314 | 0.032993401 |
| Acetazolamide | D000086 | 1.783718335 | 0.033193361 |
| Glycyrrhizic Acid | D019695 | 1.803520699 | 0.033993201 |
| Desipramine | D003891 | 1.801618706 | 0.034593081 |
| Polyphenols | D059808 | 1.857126079 | 0.034593081 |
| resorcinol | C031389 | 1.855887438 | 0.034793041 |
| ursolic acid | C005466 | 1.848623355 | 0.034993001 |
| Hydrogen Peroxide | D006861 | 1.845322568 | 0.034993001 |
| poly(propyleneimine) | C443641 | 1.825908923 | 0.035592881 |
| darinaparsin | C515055 | 1.790404415 | 0.035792841 |
| aristolochic acid I | C000228 | 1.781692524 | 0.036392721 |
| quinocetone | C502851 | 1.807678273 | 0.036392721 |
| anandamide | C078814 | 1.772025464 | 0.036592681 |
| aurapten | C105832 | 1.698238263 | 0.036592681 |
| Antimycin A | D000968 | 1.86269056 | 0.036592681 |
| Tamoxifen | D013629 | 1.762892351 | 0.036592681 |
| Anisomycin | D000841 | 1.802598132 | 0.037192561 |
| sulindac sulfide | C025462 | 1.750918982 | 0.037392521 |
| Fulvestrant | D000077267 | 1.783091831 | 0.037392521 |
| SU 6656 | C416927 | 1.721658116 | 0.037592482 |
| importazole | C568452 | 1.727820332 | 0.037792442 |
| Surface-Active Agents | D013501 | 1.79095927 | 0.037992402 |
| fluoranthene | C007738 | 1.819989819 | 0.038392322 |
| Methylnitronitrosoguanidine | D008769 | 1.79077245 | 0.039392122 |
| GW 7604 | C401858 | 1.768122339 | 0.039592082 |
| cafestol | C053400 | 1.724289235 | 0.040791842 |
| Biological Products | D001688 | 1.719996893 | 0.040791842 |
| Smoke | D012906 | 1.768088157 | 0.040791842 |
| hexamethylene bisacetamide | C014026 | 1.743299155 | 0.040991802 |
| monomethylarsonous acid | C406082 | 1.736521619 | 0.040991802 |
| rottlerin | C085746 | 1.77438808 | 0.041391722 |
| Tiopronin | D008625 | 1.693859159 | 0.041591682 |
| Ethosuximide | D005013 | 1.665885362 | 0.042391522 |
| Tritolyl Phosphates | D014317 | 1.726556042 | 0.042391522 |
| Lipid Peroxides | D008054 | 1.687105222 | 0.043991202 |
| bathocuproine sulfonate | C028559 | 1.733632374 | 0.044391122 |
| C646 compound | C584509 | 1.684619719 | 0.044391122 |
| demethoxycurcumin | C050229 | 1.681677172 | 0.045190962 |
| tricarbonyldichlororuthenium (II) dimer | C447082 | 1.662080422 | 0.045190962 |
| Isoflavones | D007529 | 1.719055118 | 0.045190962 |
| Ketamine | D007649 | 1.712894702 | 0.045790842 |
| Pyocyanine | D011710 | 1.644295909 | 0.045990802 |
| Trientine | D014266 | 1.668112572 | 0.046390722 |
| ferric citrate | C025314 | 1.643337569 | 0.046790642 |
| dactolisib | C531198 | 1.675596342 | 0.046990602 |
| Tetrachloroethylene | D013750 | 1.704707203 | 0.047390522 |
| Medroxyprogesterone Acetate | D017258 | 1.669993493 | 0.047790442 |
| boric acid | C032688 | 1.621380499 | 0.048590282 |
| Fluorouracil | D005472 | 1.663025593 | 0.048590282 |
| Temozolomide | D000077204 | 1.68407921 | 0.049190162 |
| Cholecalciferol | D002762 | 1.671683517 | 0.049190162 |
| 5-hydroxythalidomide | C544471 | 1.628364472 | 0.049590082 |
| Oleanolic Acid | D009828 | 1.648854559 | 0.049790042 |
| Trientine | D014266 | 2.541201019 | 0.00319936 |
| Vitallium | D014800 | 2.479437497 | 0.00519896 |
| Soman | D012999 | 2.370659668 | 0.00939812 |
| Coal Ash | D060729 | 2.293967354 | 0.00939812 |
| potassium perchlorate | C009006 | 2.406286239 | 0.00979804 |
| daidzin | C013908 | 2.136205132 | 0.013397321 |
| Chenodeoxycholic Acid | D002635 | 2.161705275 | 0.013397321 |
| glycitein | C086566 | 2.112323762 | 0.014197161 |
| Antirheumatic Agents | D018501 | 2.191864122 | 0.015396921 |
| fenoxycarb | C052034 | 2.055546652 | 0.015796841 |
| Mycophenolic Acid | D009173 | 2.100801779 | 0.016196761 |
| Dexrazoxane | D064730 | 1.995652238 | 0.021795641 |
| Fenretinide | D017313 | 2.051099197 | 0.023195361 |
| Mevalonic Acid | D008798 | 1.959600458 | 0.023595281 |
| Taurocholic Acid | D013656 | 1.885646161 | 0.024395121 |
| Levofloxacin | D064704 | 1.975441723 | 0.024795041 |
| Naphthoquinones | D009285 | 1.93106266 | 0.026794641 |
| Rotenone | D012402 | 1.954810582 | 0.026794641 |
| Dextran Sulfate | D016264 | 1.987687226 | 0.026794641 |
| 2-chloroethyl ethyl sulfide | C031278 | 1.825368868 | 0.029394121 |
| Cholic Acids | D002793 | 1.809173106 | 0.031993601 |
| RTKI cpd | C101044 | 1.781288382 | 0.033593281 |
| PD 0325901 | C506614 | 1.742219209 | 0.034793041 |
| CD 437 | C099555 | 1.754152222 | 0.036592681 |
| Calcium Chloride | D002122 | 1.761392831 | 0.036992601 |
| genistin | C040641 | 1.730989526 | 0.039392122 |
| perfluorododecanoic acid | C522391 | 1.723812325 | 0.040991802 |
| Sterigmatocystin | D013241 | 1.689771711 | 0.042791442 |
| Calcium | D002118 | 1.738935201 | 0.044191162 |
| Griseofulvin | D006118 | 1.707878456 | 0.045790842 |
| heliotrine | C010178 | 1.680235193 | 0.046390722 |
| Arsenic | D001151 | 1.712316649 | 0.046990602 |
| tris(2-butoxyethyl) phosphate | C013320 | 1.711973878 | 0.047190562 |
| testosterone-3-carboxymethyloxime-bovine serum albumin conjugate | C045037 | 1.633202896 | 0.048190362 |

^1^ Chemical ID: The ID of the chemicals in [Comparative Toxicogenomics Database](http://ctdbase.org/)(CTD).

^2^ NES: Normalized enrichment score.

## Supplementary Table 2. 103 significant chemicals related to GWAS summary datasets of rectal cancer. (P value＜0.05)

| Chemical Name | Chemical ID^1^ | NES^2^ | P value |
| --- | --- | --- | --- |
| lupeol | C010480 | 2.544626494 | 0.00159968 |
| ethinyl estradiol-desogestrel combination | C442659 | 2.705765012 | 0.00179964 |
| chrysophanic acid | C027113 | 2.67336359 | 0.00259948 |
| Polyethyleneimine | D011094 | 2.650559978 | 0.0029994 |
| Mitotane | D008939 | 2.752243607 | 0.00339932 |
| etomoxir | C054207 | 2.439751753 | 0.00379924 |
| vitexin | C032731 | 2.384310967 | 0.004999 |
| sulindac sulfide | C025462 | 2.553718045 | 0.00519896 |
| SDZ FOX 988 | C094089 | 2.356875671 | 0.00519896 |
| Proanthocyanidins | D044945 | 2.433394535 | 0.00579884 |
| Chlorambucil | D002699 | 2.522925084 | 0.00639872 |
| tert-Butyl Alcohol | D020002 | 2.270994416 | 0.00779844 |
| Alendronate | D019386 | 2.370759526 | 0.00859828 |
| Brefeldin A | D020126 | 2.392726803 | 0.00859828 |
| Thiostrepton | D013883 | 2.407745203 | 0.00879824 |
| Fenitrothion | D005278 | 2.218537819 | 0.00959808 |
| Amsacrine | D000677 | 2.220539425 | 0.01079784 |
| UF010 compound | C000602704 | 2.221850576 | 0.01119776 |
| Ethosuximide | D005013 | 2.088372324 | 0.01239752 |
| bakuchiol | C012765 | 2.215264503 | 0.012597481 |
| Methylnitronitrosoguanidine | D008769 | 2.289874973 | 0.013197361 |
| afimoxifene | C016601 | 2.296924572 | 0.013397321 |
| Semaxinib | C116890 | 2.046170086 | 0.013797241 |
| chloroacetic acid | C006972 | 2.164766202 | 0.014797041 |
| Cytotoxins | D003603 | 2.064114402 | 0.016996601 |
| Tocopherols | D024505 | 2.085110195 | 0.016996601 |
| Nickel | D009532 | 2.105072089 | 0.017796441 |
| ochratoxin A | C025589 | 2.091291655 | 0.017996401 |
| fusarenon-X | C002469 | 2.003528363 | 0.018396321 |
| Linoleic Acid | D019787 | 2.117730656 | 0.018396321 |
| paricalcitol | C084656 | 2.12948806 | 0.018996201 |
| Orlistat | D000077403 | 2.131308074 | 0.019596081 |
| Promethazine | D011398 | 1.991797113 | 0.019596081 |
| Aldosterone | D000450 | 2.049059527 | 0.019796041 |
| methyleugenol | C005223 | 2.030664412 | 0.020195961 |
| pyrimidin-2-one beta-ribofuranoside | C009131 | 2.010755046 | 0.020595881 |
| Probucol | D011341 | 1.990540082 | 0.020595881 |
| gambogic acid | C052659 | 1.973498766 | 0.021995601 |
| ML 9 | C056218 | 1.950463038 | 0.022595481 |
| Levetiracetam | D000077287 | 1.90618902 | 0.023795241 |
| Ethylmaleimide | D005033 | 1.961047294 | 0.023795241 |
| Mercaptopurine | D015122 | 2.026405171 | 0.023995201 |
| Quetiapine Fumarate | D000069348 | 1.941766649 | 0.024395121 |
| Thapsigargin | D019284 | 1.932255625 | 0.024395121 |
| Isoniazid | D007538 | 1.928859829 | 0.026194761 |
| 8-oxo-7-hydrodeoxyguanosine | C067134 | 1.885870866 | 0.026794641 |
| ethoxyacetic acid | C034096 | 1.83344866 | 0.026994601 |
| apple polyphenol extract | C526219 | 1.887785957 | 0.027394521 |
| incobotulinumtoxinA | C545476 | 1.969276639 | 0.027594481 |
| 4-anisidine | C013813 | 1.878367895 | 0.028394321 |
| Poly(amidoamine) | C531249 | 1.872610361 | 0.028594281 |
| Retinaldehyde | D012172 | 1.87648621 | 0.030393921 |
| 2-aminofluorene | C012177 | 1.822946789 | 0.030793841 |
| Cycloheximide | D003513 | 1.917881365 | 0.030993801 |
| Amino Acids | D000596 | 1.850353766 | 0.031193761 |
| zearalenol | C029659 | 1.823925774 | 0.032593481 |
| usnic acid | C073339 | 1.808840109 | 0.034393121 |
| 3-(5'-hydroxymethyl-2'-furyl)-1-benzylindazole | C090937 | 1.766169692 | 0.034593081 |
| CEP-11004 | C469298 | 1.787154576 | 0.034993001 |
| bromoacetate | C016274 | 1.789937073 | 0.035592881 |
| NADP | D009249 | 1.817930344 | 0.035792841 |
| LL-202 | C000589248 | 1.764501444 | 0.037392521 |
| ON123300 | C000594117 | 1.742726632 | 0.038592282 |
| cadmium sulfide | C034939 | 1.795937309 | 0.038592282 |
| Oligomycins | D009840 | 1.749098767 | 0.038592282 |
| Mitomycin | D016685 | 1.776706783 | 0.039592082 |
| lemongrass oil | C052901 | 1.775311621 | 0.039992002 |
| CC-8490 | C495817 | 1.715755252 | 0.040991802 |
| Hydroxamic Acids | D006877 | 1.706988806 | 0.041191762 |
| danthron | C004315 | 1.710871545 | 0.041791642 |
| thermozymocidin | C001996 | 1.654532683 | 0.043391322 |
| Niacinamide | D009536 | 1.712740163 | 0.044191162 |
| N-palmitoylsphingosine | C097760 | 1.652147657 | 0.044391122 |
| importazole | C568452 | 1.681119537 | 0.044991002 |
| Quinic Acid | D011801 | 1.700698355 | 0.046190762 |
| pantogab | C016030 | 1.689387323 | 0.046390722 |
| Triamcinolone Acetonide | D014222 | 1.606866342 | 0.047190562 |
| Benomyl | D001542 | 1.647459945 | 0.047590482 |
| nodularin | C063998 | 1.666501886 | 0.048190362 |
| Cyanamide | D003484 | 1.63631984 | 0.048390322 |
| St. Thomas' Hospital cardioplegic solution | C041711 | 1.667150632 | 0.048590282 |
| 4-(3-(2-propyl-3-hydroxy-4-acetyl)phenoxy)propyloxyphenoxy acetic acid | C520164 | 1.645639628 | 0.048590282 |
| Azoxymethane | D001397 | 1.623930473 | 0.049390122 |
| fumonisin B1 | C056933 | 2.971683522 | 0.00139972 |
| NAD | D009243 | 2.684430487 | 0.0019996 |
| casticin | C054133 | 2.231423929 | 0.00859828 |
| benz(a)anthracene | C030935 | 2.264296922 | 0.01239752 |
| 4-phenylbutyric acid | C075773 | 2.095061861 | 0.014997001 |
| quercitrin | C012526 | 2.114116454 | 0.017596481 |
| 4-(2-bromo-6-pyrrolidin-1-ylpyridine-4-sulfonyl)phenylamine | C473484 | 1.990224518 | 0.020195961 |
| Ro 65-7199 | C488333 | 1.990224518 | 0.020195961 |
| beta Carotene | D019207 | 1.99633798 | 0.022595481 |
| gardiquimod | C546771 | 1.933829145 | 0.022995401 |
| Luteolin | D047311 | 1.878579225 | 0.027994401 |
| Plant Oils | D010938 | 1.848007895 | 0.032793441 |
| perfluoro-n-nonanoic acid | C101816 | 1.723977931 | 0.035992801 |
| Venlafaxine Hydrochloride | D000069470 | 1.773427544 | 0.037192561 |
| Colforsin | D005576 | 1.770014285 | 0.037592482 |
| Vancomycin | D014640 | 1.816854986 | 0.039192162 |
| PD 0325901 | C506614 | 1.68753574 | 0.040591882 |
| geldanamycin | C001277 | 1.747237117 | 0.041191762 |
| Citrinin | D002953 | 1.640266813 | 0.044591082 |
| Toxaphene | D014112 | 1.643732972 | 0.049390122 |

^1^ Chemical ID: The ID of the chemicals in [Comparative Toxicogenomics Database](http://ctdbase.org/)(CTD).

^2^ NES: Normalized enrichment score.

## Supplementary Table 3. 1198 significant chemicals related to the mRNA expression profile of colorectal cancer. (P value＜0.05)

| Chemical Name | Chemical ID^1^ | NES^2^ | P value |
| --- | --- | --- | --- |
| GSK-J4 | C000593030 | 5.507646084 | 0.00019996 |
| bisphenol F | C000611646 | 6.43569661 | 0.00019996 |
| dicrotophos | C000944 | 12.33835003 | 0.00019996 |
| triptolide | C001899 | 4.59733143 | 0.00019996 |
| N-nitrosomorpholine | C002741 | 4.24614991 | 0.00019996 |
| chloroacetaldehyde | C004656 | 4.067853593 | 0.00019996 |
| methylmercuric chloride | C004925 | 11.35972745 | 0.00019996 |
| bis(tri-n-butyltin)oxide | C005961 | 5.656539458 | 0.00019996 |
| propylparaben | C006068 | 4.089059605 | 0.00019996 |
| 2-amino-2-methyl-1-propanol | C006551 | 5.633575333 | 0.00019996 |
| benzo(b)fluoranthene | C006703 | 6.666178975 | 0.00019996 |
| deoxynivalenol | C007262 | 4.575959256 | 0.00019996 |
| fluoranthene | C007738 | 4.665718266 | 0.00019996 |
| geraniol | C007836 | 5.170786503 | 0.00019996 |
| lead acetate | C008261 | 6.544512423 | 0.00019996 |
| methylselenic acid | C008493 | 4.17959543 | 0.00019996 |
| sodium arsenate | C009277 | 6.352505819 | 0.00019996 |
| titanium dioxide | C009495 | 10.40443138 | 0.00019996 |
| decabromobiphenyl ether | C010902 | 3.801641897 | 0.00019996 |
| mezerein | C011309 | 3.436767666 | 0.00019996 |
| tributyltin | C011559 | 6.464172932 | 0.00019996 |
| kojic acid | C011890 | 3.499739761 | 0.00019996 |
| oryzalin | C012465 | 3.137011383 | 0.00019996 |
| nimesulide | C012655 | 9.974401807 | 0.00019996 |
| tris(2-butoxyethyl) phosphate | C013320 | 3.899963465 | 0.00019996 |
| bromfenacoum | C013418 | 4.779796628 | 0.00019996 |
| tallow | C013698 | 4.781028031 | 0.00019996 |
| nitrosobenzylmethylamine | C014707 | 6.022627651 | 0.00019996 |
| arsenite | C015001 | 4.818606892 | 0.00019996 |
| trimellitic anhydride | C015559 | 15.35495437 | 0.00019996 |
| sodium bichromate | C016104 | 11.70812303 | 0.00019996 |
| indole-3-carbinol | C016517 | 4.9277929 | 0.00019996 |
| 4-(N-methyl-N-nitrosamino)-1-(3-pyridyl)-1-butanone | C016583 | 4.992149687 | 0.00019996 |
| afimoxifene | C016601 | 5.957793363 | 0.00019996 |
| sulforafan | C016766 | 4.239311989 | 0.00019996 |
| zinc chloride | C016837 | 4.579526529 | 0.00019996 |
| n-butoxyethanol | C017096 | 6.008497656 | 0.00019996 |
| 3-dinitrobenzene | C017906 | 4.17175896 | 0.00019996 |
| sodium arsenite | C017947 | 10.02440593 | 0.00019996 |
| cobaltous chloride | C018021 | 16.8657461 | 0.00019996 |
| 2-nitrofluorene | C019499 | 5.398332488 | 0.00019996 |
| tetrabromobisphenol A | C020806 | 4.28430165 | 0.00019996 |
| nickel chloride | C022838 | 8.518997276 | 0.00019996 |
| perfluorooctanoic acid | C023036 | 10.47731824 | 0.00019996 |
| fludarabine | C024352 | 4.094740435 | 0.00019996 |
| nonylphenol | C025256 | 4.006398368 | 0.00019996 |
| manganese chloride | C025340 | 4.758184327 | 0.00019996 |
| sulindac sulfide | C025462 | 4.056160794 | 0.00019996 |
| ochratoxin A | C025589 | 5.454982719 | 0.00019996 |
| vinclozolin | C025643 | 10.82752897 | 0.00019996 |
| potassium chromate(VI) | C027373 | 8.242466266 | 0.00019996 |
| 4-hydroxy-2-nonenal | C027576 | 6.142268533 | 0.00019996 |
| nickel monoxide | C028007 | 5.693936127 | 0.00019996 |
| vinylidene chloride | C029297 | 6.738222372 | 0.00019996 |
| cupric chloride | C029892 | 4.330778332 | 0.00019996 |
| nickel sulfate | C029938 | 6.008229067 | 0.00019996 |
| benz(a)anthracene | C030935 | 4.125719715 | 0.00019996 |
| cupric oxide | C030973 | 6.075669375 | 0.00019996 |
| pyrazole | C031280 | 4.892622088 | 0.00019996 |
| naphthalene | C031721 | 5.85658519 | 0.00019996 |
| hydroquinone | C031927 | 4.045783874 | 0.00019996 |
| 4-aminophenylarsenoxide | C032642 | 5.125455123 | 0.00019996 |
| 1-nitropyrene | C032668 | 4.810160808 | 0.00019996 |
| dicyclohexyl phthalate | C036042 | 4.526876692 | 0.00019996 |
| leptomycin B | C038753 | 3.604590004 | 0.00019996 |
| furan | C039281 | 9.658594321 | 0.00019996 |
| ciglitazone | C039671 | 4.90099653 | 0.00019996 |
| isobutyl alcohol | C040507 | 4.04029203 | 0.00019996 |
| mercuric bromide | C042720 | 4.167302729 | 0.00019996 |
| beta-methylcholine | C044887 | 11.13722643 | 0.00019996 |
| testosterone-3-carboxymethyloxime-bovine serum albumin conjugate | C045037 | 3.583504904 | 0.00019996 |
| propiconazole | C045950 | 6.911355934 | 0.00019996 |
| pinosylvin | C049032 | 4.816587743 | 0.00019996 |
| 1-methylanthracene | C051246 | 4.145546049 | 0.00019996 |
| nefazodone | C051752 | 10.38090946 | 0.00019996 |
| rebamipide | C052785 | 3.619796871 | 0.00019996 |
| bicalutamide | C053541 | 5.233287253 | 0.00019996 |
| casticin | C054133 | 3.725559824 | 0.00019996 |
| tin mesoporphyrin | C055421 | 3.673211322 | 0.00019996 |
| gemcitabine | C056507 | 4.525635552 | 0.00019996 |
| phenethyl isothiocyanate | C058305 | 5.673340071 | 0.00019996 |
| polyhexamethyleneguanidine | C060540 | 4.914673482 | 0.00019996 |
| benzyloxycarbonylleucyl-leucyl-leucine aldehyde | C072553 | 5.297546633 | 0.00019996 |
| chromium hexavalent ion | C074702 | 12.08022787 | 0.00019996 |
| squalestatin 1 | C075117 | 5.82451169 | 0.00019996 |
| perfluorooctane sulfonic acid | C076994 | 5.373254176 | 0.00019996 |
| seocalcitol | C078903 | 4.251295041 | 0.00019996 |
| trovafloxacin | C080163 | 4.969203975 | 0.00019996 |
| 2-(4-morpholinyl)-8-phenyl-4H-1-benzopyran-4-one | C085911 | 3.929974204 | 0.00019996 |
| pentabromodiphenyl ether | C086401 | 7.348525503 | 0.00019996 |
| plerixafor octahydrochloride | C088327 | 3.37860227 | 0.00019996 |
| cylindrospermopsin | C089595 | 4.640064553 | 0.00019996 |
| JP8 aviation fuel | C098607 | 3.904643433 | 0.00019996 |
| bifenthrin | C099952 | 3.967051533 | 0.00019996 |
| chloropicrin | C100187 | 9.042647825 | 0.00019996 |
| pterostilbene | C107773 | 3.760988615 | 0.00019996 |
| monomethylarsonous acid | C406082 | 4.424172827 | 0.00019996 |
| valdecoxib | C406224 | 5.850402681 | 0.00019996 |
| CPG-oligonucleotide | C408982 | 4.237487798 | 0.00019996 |
| PCB 180 | C410127 | 5.690767978 | 0.00019996 |
| K 7174 | C410337 | 12.45309282 | 0.00019996 |
| AGN 194204 | C430898 | 4.099536895 | 0.00019996 |
| GW 7647 | C453899 | 3.750090126 | 0.00019996 |
| obeticholic acid | C464660 | 3.849927026 | 0.00019996 |
| 3-(4'-hydroxy-3'-adamantylbiphenyl-4-yl)acrylic acid | C472791 | 4.706221856 | 0.00019996 |
| ICG 001 | C492448 | 16.23322695 | 0.00019996 |
| perchlorate | C494474 | 3.570019699 | 0.00019996 |
| 2-hydroxy-9-cis-octadecenoic acid | C497101 | 2.828160022 | 0.00019996 |
| palbociclib | C500026 | 10.0585019 | 0.00019996 |
| muraglitazar | C500085 | 4.653942752 | 0.00019996 |
| tesaglitazar | C501413 | 4.52911132 | 0.00019996 |
| quinocetone | C502851 | 3.928504642 | 0.00019996 |
| ON 01910 | C507134 | 3.595883397 | 0.00019996 |
| ormosil | C510784 | 4.221942503 | 0.00019996 |
| 2-methyl-2H-pyrazole-3-carboxylic acid (2-methyl-4-o-tolylazophenyl)amide | C511621 | 5.262045705 | 0.00019996 |
| pyrachlostrobin | C513428 | 5.104524345 | 0.00019996 |
| S-2-pentyl-4-pentynoic hydroxamic acid | C513635 | 3.544616301 | 0.00019996 |
| monomethyl phthalate | C517284 | 5.539214777 | 0.00019996 |
| sandostatinLAR | C541923 | 3.480285913 | 0.00019996 |
| bis(4-hydroxyphenyl)sulfone | C543008 | 5.837207575 | 0.00019996 |
| incobotulinumtoxinA | C545476 | 9.911886354 | 0.00019996 |
| AZM551248 | C547126 | 9.513284685 | 0.00019996 |
| riccardin D | C547185 | 3.814189503 | 0.00019996 |
| 2-(1'H-indolo-3'-carbonyl)thiazole-4-carboxylic acid methyl ester | C548651 | 3.380057719 | 0.00019996 |
| NSC 689534 | C558013 | 10.85043021 | 0.00019996 |
| (+)-JQ1 compound | C561695 | 12.27771161 | 0.00019996 |
| PCI 5002 | C568608 | 4.256215671 | 0.00019996 |
| NSC668394 | C570897 | 4.494597521 | 0.00019996 |
| C646 compound | C584509 | 12.64983433 | 0.00019996 |
| Celecoxib | D000068579 | 3.833030363 | 0.00019996 |
| Bortezomib | D000069286 | 4.398642582 | 0.00019996 |
| Dasatinib | D000069439 | 10.1482673 | 0.00019996 |
| Palm Oil | D000073878 | 7.002935855 | 0.00019996 |
| Irinotecan | D000077146 | 5.97119001 | 0.00019996 |
| Oxaliplatin | D000077150 | 13.80277369 | 0.00019996 |
| Rosiglitazone | D000077154 | 8.182622547 | 0.00019996 |
| Resveratrol | D000077185 | 13.74716186 | 0.00019996 |
| Decitabine | D000077209 | 7.085223392 | 0.00019996 |
| Zoledronic Acid | D000077211 | 9.477005075 | 0.00019996 |
| Arsenic Trioxide | D000077237 | 12.18003025 | 0.00019996 |
| Fulvestrant | D000077267 | 6.426684797 | 0.00019996 |
| Rimonabant | D000077285 | 3.636570602 | 0.00019996 |
| Troglitazone | D000077288 | 11.03620345 | 0.00019996 |
| Vorinostat | D000077337 | 6.549654891 | 0.00019996 |
| Leflunomide | D000077339 | 9.701458169 | 0.00019996 |
| Silybin | D000077385 | 3.841006901 | 0.00019996 |
| Cidofovir | D000077404 | 4.074757163 | 0.00019996 |
| 2-Methoxyestradiol | D000077584 | 3.900267229 | 0.00019996 |
| Panobinostat | D000077767 | 3.809853002 | 0.00019996 |
| Tolcapone | D000077867 | 3.850795102 | 0.00019996 |
| Acetylcysteine | D000111 | 5.576265795 | 0.00019996 |
| Adenine | D000225 | 3.867857146 | 0.00019996 |
| Aerosols | D000336 | 5.187294226 | 0.00019996 |
| Air Pollutants | D000393 | 5.267673187 | 0.00019996 |
| Ethanol | D000431 | 7.355749587 | 0.00019996 |
| alpha-Chlorohydrin | D000517 | 5.035640329 | 0.00019996 |
| Amiodarone | D000638 | 4.034310481 | 0.00019996 |
| Ammonium Chloride | D000643 | 7.476110976 | 0.00019996 |
| Anthralin | D000875 | 3.899760447 | 0.00019996 |
| Antimony | D000965 | 3.703023683 | 0.00019996 |
| Antimony Potassium Tartrate | D000966 | 3.664986214 | 0.00019996 |
| Arsenic | D001151 | 7.771464318 | 0.00019996 |
| Ascorbic Acid | D001205 | 6.958171724 | 0.00019996 |
| Aspirin | D001241 | 4.446284135 | 0.00019996 |
| Azacitidine | D001374 | 3.153339533 | 0.00019996 |
| Azathioprine | D001379 | 10.42146729 | 0.00019996 |
| Beclomethasone | D001507 | 4.352124634 | 0.00019996 |
| Benzbromarone | D001553 | 3.742859954 | 0.00019996 |
| Benzene | D001554 | 4.412159552 | 0.00019996 |
| Hexachlorocyclohexane | D001556 | 4.829699369 | 0.00019996 |
| Betaine | D001622 | 4.459213843 | 0.00019996 |
| Bezafibrate | D001629 | 4.561671177 | 0.00019996 |
| Buspirone | D002065 | 4.924385462 | 0.00019996 |
| Butylated Hydroxyanisole | D002083 | 3.510587693 | 0.00019996 |
| Butyrates | D002087 | 4.43196448 | 0.00019996 |
| Cadmium | D002104 | 8.598040287 | 0.00019996 |
| Caffeine | D002110 | 3.777961394 | 0.00019996 |
| Calcitriol | D002117 | 15.03337056 | 0.00019996 |
| Capsaicin | D002211 | 4.083460454 | 0.00019996 |
| Carbamazepine | D002220 | 9.074778941 | 0.00019996 |
| Carbon Tetrachloride | D002251 | 13.41280367 | 0.00019996 |
| Carbonyl Cyanide p-Trifluoromethoxyphenylhydrazone | D002259 | 3.731968692 | 0.00019996 |
| Cephaloridine | D002509 | 7.036271274 | 0.00019996 |
| Chloroprene | D002737 | 4.354951413 | 0.00019996 |
| Choline | D002794 | 9.542900371 | 0.00019996 |
| Ciguatoxins | D002922 | 4.226627901 | 0.00019996 |
| Clofibrate | D002994 | 9.223393705 | 0.00019996 |
| Clofibric Acid | D002995 | 7.741625131 | 0.00019996 |
| Cobalt | D003035 | 4.136341479 | 0.00019996 |
| Colchicine | D003078 | 4.615063131 | 0.00019996 |
| Copper | D003300 | 12.07016805 | 0.00019996 |
| Corticosterone | D003345 | 5.836566242 | 0.00019996 |
| Coumestrol | D003375 | 16.18097277 | 0.00019996 |
| Croton Oil | D003436 | 4.438542838 | 0.00019996 |
| Cuprizone | D003471 | 4.820364545 | 0.00019996 |
| Curcumin | D003474 | 5.761610128 | 0.00019996 |
| Cyclophosphamide | D003520 | 4.969169618 | 0.00019996 |
| Cytarabine | D003561 | 4.464188262 | 0.00019996 |
| Dichlorodiphenyl Dichloroethylene | D003633 | 3.744853954 | 0.00019996 |
| Demecolcine | D003703 | 5.586889547 | 0.00019996 |
| Diazinon | D003976 | 5.439533879 | 0.00019996 |
| Dibutyl Phthalate | D003993 | 15.54171537 | 0.00019996 |
| Dichloroacetic Acid | D003999 | 5.007155825 | 0.00019996 |
| Diclofenac | D004008 | 4.718602374 | 0.00019996 |
| Dieldrin | D004026 | 6.730585025 | 0.00019996 |
| Succimer | D004113 | 4.858143975 | 0.00019996 |
| Chlorpyrifos | D004390 | 4.936900574 | 0.00019996 |
| Estrogens | D004967 | 5.484138713 | 0.00019996 |
| Ethyl Methanesulfonate | D005020 | 9.969566733 | 0.00019996 |
| Etoposide | D005047 | 7.48230294 | 0.00019996 |
| Flavonoids | D005419 | 4.685588022 | 0.00019996 |
| Fluorides | D005459 | 3.51333669 | 0.00019996 |
| Fluorouracil | D005472 | 9.44314862 | 0.00019996 |
| Flutamide | D005485 | 16.26944813 | 0.00019996 |
| Folic Acid | D005492 | 10.74518634 | 0.00019996 |
| Formaldehyde | D005557 | 10.57336699 | 0.00019996 |
| Gasoline | D005742 | 9.24760783 | 0.00019996 |
| Gentamicins | D005839 | 9.098475898 | 0.00019996 |
| Glafenine | D005897 | 7.740485831 | 0.00019996 |
| Glucosamine | D005944 | 4.955939094 | 0.00019996 |
| Glucose | D005947 | 7.912040168 | 0.00019996 |
| Glutathione | D005978 | 4.786029613 | 0.00019996 |
| Gold | D006046 | 4.240842862 | 0.00019996 |
| Heptachlor | D006533 | 3.251920104 | 0.00019996 |
| Hyaluronic Acid | D006820 | 3.522418693 | 0.00019996 |
| Hydrogen Peroxide | D006861 | 13.6309904 | 0.00019996 |
| Hydroxyurea | D006918 | 3.889344248 | 0.00019996 |
| Hypochlorous Acid | D006997 | 3.951149208 | 0.00019996 |
| Indomethacin | D007213 | 13.64272546 | 0.00019996 |
| Iron | D007501 | 3.724377875 | 0.00019996 |
| Isoproterenol | D007545 | 4.63408041 | 0.00019996 |
| Ketamine | D007649 | 5.12074543 | 0.00019996 |
| Latex | D007840 | 6.793323396 | 0.00019996 |
| Lead | D007854 | 7.325660073 | 0.00019996 |
| Lipopolysaccharides | D008070 | 6.640214989 | 0.00019996 |
| Lovastatin | D008148 | 4.044852556 | 0.00019996 |
| Lucanthone | D008154 | 8.093821364 | 0.00019996 |
| Mercuric Chloride | D008627 | 6.040111131 | 0.00019996 |
| Mercury | D008628 | 3.718495477 | 0.00019996 |
| Mestranol | D008656 | 3.204751126 | 0.00019996 |
| Metformin | D008687 | 4.737161416 | 0.00019996 |
| Methamphetamine | D008694 | 10.79769873 | 0.00019996 |
| Methapyrilene | D008701 | 6.694506102 | 0.00019996 |
| Methionine | D008715 | 9.538905407 | 0.00019996 |
| Methotrexate | D008727 | 7.900343549 | 0.00019996 |
| Methylcholanthrene | D008748 | 9.279674954 | 0.00019996 |
| Methylmercury Compounds | D008767 | 6.461334357 | 0.00019996 |
| Methylnitronitrosoguanidine | D008769 | 3.735167752 | 0.00019996 |
| Methylnitrosourea | D008770 | 4.024387903 | 0.00019996 |
| Methyltestosterone | D008777 | 7.015748334 | 0.00019996 |
| Mitoxantrone | D008942 | 3.688343609 | 0.00019996 |
| Mustard Gas | D009151 | 9.026589778 | 0.00019996 |
| Nickel | D009532 | 12.37649043 | 0.00019996 |
| Niclosamide | D009534 | 5.312581493 | 0.00019996 |
| Oxazolone | D010081 | 5.43088582 | 0.00019996 |
| Oxygen | D010100 | 14.97978109 | 0.00019996 |
| Ozone | D010126 | 7.57819866 | 0.00019996 |
| Paraquat | D010269 | 6.848342904 | 0.00019996 |
| Pentachlorophenol | D010416 | 8.65444942 | 0.00019996 |
| Phenobarbital | D010634 | 15.82457188 | 0.00019996 |
| Phenylephrine | D010656 | 4.865842154 | 0.00019996 |
| Phenylmercuric Acetate | D010662 | 7.015158619 | 0.00019996 |
| Piperonyl Butoxide | D010882 | 5.143164343 | 0.00019996 |
| Piroxicam | D010894 | 9.207387915 | 0.00019996 |
| Plant Extracts | D010936 | 4.857361592 | 0.00019996 |
| Polychlorinated Biphenyls | D011078 | 7.364701944 | 0.00019996 |
| Polycyclic Aromatic Hydrocarbons | D011084 | 9.827943842 | 0.00019996 |
| Polyethylene Glycols | D011092 | 5.022286575 | 0.00019996 |
| Potassium Dichromate | D011192 | 5.956329744 | 0.00019996 |
| Pregnenolone Carbonitrile | D011285 | 9.818157442 | 0.00019996 |
| Fenofibrate | D011345 | 6.471038233 | 0.00019996 |
| Progestins | D011372 | 3.795386621 | 0.00019996 |
| Progesterone | D011374 | 12.42214138 | 0.00019996 |
| Propylthiouracil | D011441 | 9.062891614 | 0.00019996 |
| Quercetin | D011794 | 12.80138911 | 0.00019996 |
| Rotenone | D012402 | 6.756841096 | 0.00019996 |
| Selenium | D012643 | 6.474138783 | 0.00019996 |
| Sesame Oil | D012715 | 4.243744705 | 0.00019996 |
| Silicon Dioxide | D012822 | 12.85106548 | 0.00019996 |
| Silver | D012834 | 6.987921384 | 0.00019996 |
| Silver Nitrate | D012835 | 4.89810703 | 0.00019996 |
| Smoke | D012906 | 4.35922067 | 0.00019996 |
| Soman | D012999 | 7.244134432 | 0.00019996 |
| Streptozocin | D013311 | 4.01809151 | 0.00019996 |
| Sulindac | D013467 | 4.170349027 | 0.00019996 |
| T-2 Toxin | D013605 | 5.10453795 | 0.00019996 |
| Tamoxifen | D013629 | 10.30168212 | 0.00019996 |
| Testosterone | D013739 | 13.45224234 | 0.00019996 |
| Tetrachloroethylene | D013750 | 5.07884132 | 0.00019996 |
| Tetradecanoylphorbol Acetate | D013755 | 17.49864392 | 0.00019996 |
| Thioacetamide | D013853 | 11.30409228 | 0.00019996 |
| Thiram | D013893 | 4.697999169 | 0.00019996 |
| Trichloroethylene | D014241 | 7.230495543 | 0.00019996 |
| Triclosan | D014260 | 5.936798827 | 0.00019996 |
| Triiodothyronine | D014284 | 3.354579538 | 0.00019996 |
| Trinitrobenzenesulfonic Acid | D014302 | 3.639436179 | 0.00019996 |
| Trinitrotoluene | D014303 | 5.330023747 | 0.00019996 |
| Tunicamycin | D014415 | 11.94143273 | 0.00019996 |
| Urethane | D014520 | 10.06711158 | 0.00019996 |
| Vanadates | D014638 | 4.861140676 | 0.00019996 |
| Vancomycin | D014640 | 3.699502246 | 0.00019996 |
| Vinblastine | D014747 | 6.525581393 | 0.00019996 |
| Vincristine | D014750 | 8.167423511 | 0.00019996 |
| Vitallium | D014800 | 5.319800219 | 0.00019996 |
| Vitamin E | D014810 | 6.623930823 | 0.00019996 |
| Zearalenone | D015025 | 4.945169766 | 0.00019996 |
| Zinc | D015032 | 6.907407994 | 0.00019996 |
| 1-Methyl-3-isobutylxanthine | D015056 | 11.50615314 | 0.00019996 |
| 1-Naphthylisothiocyanate | D015058 | 5.54737294 | 0.00019996 |
| 2-Acetylaminofluorene | D015073 | 4.601857836 | 0.00019996 |
| Mercaptopurine | D015122 | 3.900074273 | 0.00019996 |
| Zidovudine | D015215 | 4.727623679 | 0.00019996 |
| Isotretinoin | D015474 | 4.536946352 | 0.00019996 |
| 1-Methyl-4-phenylpyridinium | D015655 | 8.256149983 | 0.00019996 |
| Mifepristone | D015735 | 6.907174178 | 0.00019996 |
| Metribolone | D015741 | 4.675729494 | 0.00019996 |
| Ionomycin | D015759 | 16.80540634 | 0.00019996 |
| Tacrolimus | D016559 | 4.320916282 | 0.00019996 |
| Mitomycin | D016685 | 5.731968188 | 0.00019996 |
| Pravastatin | D017035 | 4.242120193 | 0.00019996 |
| Paclitaxel | D017239 | 6.102910888 | 0.00019996 |
| Fenretinide | D017313 | 12.68077767 | 0.00019996 |
| Nitric Acid | D017942 | 2.903893445 | 0.00019996 |
| Tungsten Compounds | D017973 | 4.268431901 | 0.00019996 |
| Sodium Selenite | D018038 | 8.314763559 | 0.00019996 |
| Finasteride | D018120 | 6.434241442 | 0.00019996 |
| Antirheumatic Agents | D018501 | 6.506736828 | 0.00019996 |
| Cadmium Chloride | D019256 | 13.17684306 | 0.00019996 |
| Lamivudine | D019259 | 4.506519687 | 0.00019996 |
| Thapsigargin | D019284 | 9.608383628 | 0.00019996 |
| Okadaic Acid | D019319 | 3.55109843 | 0.00019996 |
| beta-Naphthoflavone | D019324 | 5.202867233 | 0.00019996 |
| Buthionine Sulfoximine | D019328 | 4.760604591 | 0.00019996 |
| Ritonavir | D019438 | 3.913358207 | 0.00019996 |
| Topotecan | D019772 | 14.2717633 | 0.00019996 |
| Genistein | D019833 | 11.56218531 | 0.00019996 |
| 1-Butanol | D020001 | 5.923188368 | 0.00019996 |
| Acrylamide | D020106 | 7.076941691 | 0.00019996 |
| Chlorodiphenyl (54% Chlorine) | D020111 | 6.582610074 | 0.00019996 |
| tert-Butylhydroperoxide | D020122 | 9.881303299 | 0.00019996 |
| Sirolimus | D020123 | 4.748839089 | 0.00019996 |
| Butyric Acid | D020148 | 3.531866024 | 0.00019996 |
| Raloxifene Hydrochloride | D020849 | 6.147823536 | 0.00019996 |
| Vitamin K 3 | D024483 | 8.684553353 | 0.00019996 |
| Phytoestrogens | D048789 | 3.567151962 | 0.00019996 |
| Particulate Matter | D052638 | 11.71053295 | 0.00019996 |
| Soot | D053260 | 8.443714522 | 0.00019996 |
| Magnetite Nanoparticles | D058185 | 5.336872368 | 0.00019996 |
| metanil yellow | C005108 | 2.894665885 | 0.00039992 |
| propionaldehyde | C005556 | 3.592220579 | 0.00039992 |
| pseudocumene | C010313 | 3.336502678 | 0.00039992 |
| propiverine | C015586 | 3.349758912 | 0.00039992 |
| pantogab | C016030 | 3.703391281 | 0.00039992 |
| 1-nitronaphthalene | C016614 | 3.158227703 | 0.00039992 |
| cryptolepine | C024015 | 3.604208777 | 0.00039992 |
| diallyl phthalate | C049098 | 3.300460244 | 0.00039992 |
| lemongrass oil | C052901 | 4.008718941 | 0.00039992 |
| acetovanillone | C056165 | 3.453958296 | 0.00039992 |
| fullerene C60 | C069837 | 3.283295123 | 0.00039992 |
| glycidamide | C071834 | 3.701620137 | 0.00039992 |
| 4-phenylbutyric acid | C075773 | 3.361958685 | 0.00039992 |
| LG 100815 | C533894 | 3.348365776 | 0.00039992 |
| kuguacin J | C572783 | 3.120870432 | 0.00039992 |
| Bexarotene | D000077610 | 3.905057766 | 0.00039992 |
| Androstanols | D000732 | 3.353061699 | 0.00039992 |
| Cacodylic Acid | D002101 | 3.868583942 | 0.00039992 |
| Chlorpromazine | D002746 | 3.987185829 | 0.00039992 |
| Dactinomycin | D003609 | 3.319492865 | 0.00039992 |
| Ethionine | D005001 | 3.624433707 | 0.00039992 |
| Gold Sodium Thiomalate | D006052 | 3.831464639 | 0.00039992 |
| Ifosfamide | D007069 | 3.865252092 | 0.00039992 |
| Lithium | D008094 | 4.190663738 | 0.00039992 |
| Manganese | D008345 | 3.7818735 | 0.00039992 |
| NAD | D009243 | 3.517521467 | 0.00039992 |
| Naphthalenes | D009281 | 3.308320778 | 0.00039992 |
| Nicotine | D009538 | 3.849185447 | 0.00039992 |
| Petroleum | D010578 | 3.680041223 | 0.00039992 |
| Tetracycline | D013752 | 3.544913969 | 0.00039992 |
| 4-Nitroquinoline-1-oxide | D015112 | 3.399200959 | 0.00039992 |
| Medroxyprogesterone Acetate | D017258 | 4.220948542 | 0.00039992 |
| Oleic Acid | D019301 | 3.794256017 | 0.00039992 |
| Zinc Acetate | D019345 | 3.772735191 | 0.00039992 |
| Budesonide | D019819 | 3.718060406 | 0.00039992 |
| p-Chloromercuribenzoic Acid | D020245 | 3.298684755 | 0.00039992 |
| ferric oxide | C000499 | 3.012332372 | 0.00059988 |
| thymoquinone | C003466 | 3.199838762 | 0.00059988 |
| pyrrolidine dithiocarbamic acid | C020972 | 3.41109717 | 0.00059988 |
| undecane | C022884 | 3.374088807 | 0.00059988 |
| cerous chloride | C026690 | 3.417439929 | 0.00059988 |
| lead chloride | C029891 | 3.505119665 | 0.00059988 |
| coumarin | C030123 | 3.52362852 | 0.00059988 |
| usnic acid | C073339 | 3.134492009 | 0.00059988 |
| CD 437 | C099555 | 3.268485708 | 0.00059988 |
| AM 251 | C103505 | 3.26094413 | 0.00059988 |
| motexafin gadolinium | C437683 | 3.416432376 | 0.00059988 |
| 3-(4-(4-((1-(2-chlorophenyl)ethoxy)carbonyl amino)-3-methyl-5-isoxazolyl) benzylsulfanyl) propanoic acid | C477898 | 2.871024781 | 0.00059988 |
| UVI 3003 | C525131 | 3.340955458 | 0.00059988 |
| sparstolonin B | C561322 | 2.996764683 | 0.00059988 |
| 1-(4-(1-((E)-4-cyclohexyl-3-trifluoromethylbenzyloxyimino)-ethyl)-2-ethylbenzyl)-azetidine-3-carboxylic acid | C578989 | 3.389394306 | 0.00059988 |
| Aminoglutethimide | D000616 | 3.2432393 | 0.00059988 |
| Azoxymethane | D001397 | 3.118737582 | 0.00059988 |
| Chlordan | D002706 | 3.007966417 | 0.00059988 |
| Clodronic Acid | D004002 | 3.610988693 | 0.00059988 |
| Ibuprofen | D007052 | 3.26449871 | 0.00059988 |
| Maneb | D008344 | 3.28019154 | 0.00059988 |
| Phlorhizin | D010695 | 3.692933003 | 0.00059988 |
| Promethazine | D011398 | 3.166633685 | 0.00059988 |
| Pyrazinamide | D011718 | 3.379384373 | 0.00059988 |
| Reactive Oxygen Species | D017382 | 3.652058525 | 0.00059988 |
| Arsenites | D018053 | 3.219175989 | 0.00059988 |
| Ketolides | D048628 | 3.271454654 | 0.00059988 |
| naringin | C005274 | 3.236315457 | 0.00079984 |
| baicalein | C006680 | 3.487485595 | 0.00079984 |
| methotrexate polyglutamate | C014085 | 2.717856546 | 0.00079984 |
| phorone | C018637 | 3.48819265 | 0.00079984 |
| periodate-oxidized adenosine | C027579 | 3.589458432 | 0.00079984 |
| 2-chloroethyl ethyl sulfide | C031278 | 3.129136716 | 0.00079984 |
| St. Thomas' Hospital cardioplegic solution | C041711 | 3.263338988 | 0.00079984 |
| tripterine | C050414 | 3.223296446 | 0.00079984 |
| 2-(2-amino-3-methoxyphenyl)-4H-1-benzopyran-4-one | C093973 | 3.401842258 | 0.00079984 |
| aurapten | C105832 | 2.984324963 | 0.00079984 |
| gedunin | C106014 | 3.008222114 | 0.00079984 |
| oxidized-L-alpha-1-palmitoyl-2-arachidonoyl-sn-glycero-3-phosphorylcholine | C472349 | 3.401779546 | 0.00079984 |
| GW0742 | C479979 | 3.123634469 | 0.00079984 |
| bromovanin | C515564 | 3.328228609 | 0.00079984 |
| GSK1210151A | C568713 | 3.151121606 | 0.00079984 |
| N-((5-(3-(1-benzylpiperidin-4-yl)propoxy)-1-methyl-1H-indol-2-yl)methyl)-N-methylprop-2-yn-1-amine | C570530 | 2.964631509 | 0.00079984 |
| Bicarbonates | D001639 | 2.920990055 | 0.00079984 |
| Bromates | D001959 | 2.696675905 | 0.00079984 |
| Carbofuran | D002235 | 3.62175569 | 0.00079984 |
| Ciprofloxacin | D002939 | 3.13261897 | 0.00079984 |
| Cocaine | D003042 | 3.142223895 | 0.00079984 |
| Harmine | D006247 | 2.940507538 | 0.00079984 |
| Chlordecone | D007631 | 3.460234907 | 0.00079984 |
| Lithocholic Acid | D008095 | 3.082579147 | 0.00079984 |
| Methoxychlor | D008731 | 3.35684919 | 0.00079984 |
| Omeprazole | D009853 | 3.509456305 | 0.00079984 |
| Riboflavin | D012256 | 3.219986292 | 0.00079984 |
| Uranium Compounds | D017974 | 3.12867105 | 0.00079984 |
| Simvastatin | D019821 | 3.196018267 | 0.00079984 |
| Apigenin | D047310 | 3.203542869 | 0.00079984 |
| kaempferol | C006552 | 3.025288473 | 0.0009998 |
| cobaltiprotoporphyrin | C007095 | 3.094346528 | 0.0009998 |
| butyraldehyde | C018475 | 3.077780828 | 0.0009998 |
| butylidenephthalide | C026105 | 2.949074083 | 0.0009998 |
| lysophosphatidic acid | C032881 | 3.064182137 | 0.0009998 |
| microcystin RR | C063855 | 3.063582963 | 0.0009998 |
| alvocidib | C077990 | 3.022712938 | 0.0009998 |
| U 0126 | C113580 | 3.201393457 | 0.0009998 |
| SC 560 | C115461 | 2.817991247 | 0.0009998 |
| azaspiracid | C406592 | 3.024424692 | 0.0009998 |
| boldine methine | C506830 | 2.514327863 | 0.0009998 |
| MRK 003 | C523799 | 3.213194599 | 0.0009998 |
| Rapeseed Oil | D000074262 | 3.14196989 | 0.0009998 |
| Daunorubicin | D003630 | 2.828421981 | 0.0009998 |
| Hexestrol | D006589 | 2.978205188 | 0.0009998 |
| Methomyl | D008724 | 2.872160948 | 0.0009998 |
| Sodium Nitrite | D012977 | 3.006550024 | 0.0009998 |
| Ursodeoxycholic Acid | D014580 | 3.219579628 | 0.0009998 |
| malachite green | C005095 | 2.894875276 | 0.00119976 |
| salinomycin | C010327 | 3.229506418 | 0.00119976 |
| potassium bromate | C019536 | 3.18703369 | 0.00119976 |
| tanshinone | C021751 | 2.833184492 | 0.00119976 |
| caffeic acid | C040048 | 3.131848585 | 0.00119976 |
| 4-hydroxy-equilenin | C065250 | 3.072319343 | 0.00119976 |
| goniothalamin | C103280 | 3.058940424 | 0.00119976 |
| PD 0325901 | C506614 | 2.861421046 | 0.00119976 |
| 3-(2-hydroxy-4-(2-methylnonan-2-yl)phenyl)cyclohexan-1-ol | C572491 | 2.979151136 | 0.00119976 |
| Coal Tar | D003033 | 3.154788655 | 0.00119976 |
| Deferoxamine | D003676 | 3.276827894 | 0.00119976 |
| Fish Oils | D005395 | 3.041334858 | 0.00119976 |
| Hydralazine | D006830 | 3.047252391 | 0.00119976 |
| Sarin | D012524 | 3.039546556 | 0.00119976 |
| benzo(k)fluoranthene | C022921 | 2.947803312 | 0.00139972 |
| N-(2-cyclohexyloxy-4-nitrophenyl)methanesulfonamide | C080955 | 3.015484266 | 0.00139972 |
| Imatinib Mesylate | D000068877 | 2.893790335 | 0.00139972 |
| Acetaldehyde | D000079 | 2.985947223 | 0.00139972 |
| Methanol | D000432 | 2.906424488 | 0.00139972 |
| Cholic Acids | D002793 | 2.863242944 | 0.00139972 |
| Thiostrepton | D013883 | 2.782781514 | 0.00139972 |
| Lithium Chloride | D018021 | 2.984215145 | 0.00139972 |
| Silver Compounds | D018030 | 2.96930838 | 0.00139972 |
| Benzoic Acid | D019817 | 2.801191555 | 0.00139972 |
| alpha-Tocopherol | D024502 | 2.848528367 | 0.00139972 |
| aristolochic acid I | C000228 | 2.846423844 | 0.00159968 |
| fisetin | C017875 | 2.883269107 | 0.00159968 |
| cadmium acetate | C028031 | 3.049988689 | 0.00159968 |
| ferrous chloride | C029451 | 2.768301653 | 0.00159968 |
| diallyl trisulfide | C042577 | 2.945155241 | 0.00159968 |
| zerumbone | C403304 | 3.062494486 | 0.00159968 |
| Carbonates | D002254 | 2.585888526 | 0.00159968 |
| Eugenol | D005054 | 2.890053913 | 0.00159968 |
| Sulfonamides | D013449 | 2.903337387 | 0.00159968 |
| indium trichloride | C020758 | 2.731279181 | 0.00179964 |
| chrysene | C031180 | 3.25932204 | 0.00179964 |
| tauroursodeoxycholic acid | C031655 | 3.040904809 | 0.00179964 |
| cyanoginosin LR | C057862 | 3.091730792 | 0.00179964 |
| deguelin | C107676 | 3.04447162 | 0.00179964 |
| HS 1030 | C403899 | 2.511604347 | 0.00179964 |
| 6-OH-BDE-47 | C580599 | 2.916279154 | 0.00179964 |
| Wortmannin | D000077191 | 2.871723996 | 0.00179964 |
| Cycloheximide | D003513 | 2.907226662 | 0.00179964 |
| Dehydroepiandrosterone | D003687 | 2.977065648 | 0.00179964 |
| Mycophenolic Acid | D009173 | 2.930743115 | 0.00179964 |
| Niacinamide | D009536 | 2.887496877 | 0.00179964 |
| Phosgene | D010705 | 3.046703096 | 0.00179964 |
| Sodium Fluoride | D012969 | 3.040296231 | 0.00179964 |
| Thiophenes | D013876 | 2.961303442 | 0.00179964 |
| Organoselenium Compounds | D016566 | 2.869009471 | 0.00179964 |
| palytoxin | C010272 | 2.736554368 | 0.0019996 |
| ferric chloride | C024555 | 2.841270793 | 0.0019996 |
| arsenic acid | C025657 | 2.624087967 | 0.0019996 |
| oligomycin A | C031004 | 2.532881727 | 0.0019996 |
| SB 203580 | C093642 | 2.894377214 | 0.0019996 |
| Adenosine Triphosphate | D000255 | 2.74614439 | 0.0019996 |
| Butylated Hydroxytoluene | D002084 | 3.059002405 | 0.0019996 |
| Chromium | D002857 | 3.125934346 | 0.0019996 |
| Norepinephrine | D009638 | 2.871867174 | 0.0019996 |
| quinone | C004532 | 2.728739283 | 0.00219956 |
| styrene oxide | C013690 | 2.697149987 | 0.00219956 |
| chloric acid | C022842 | 2.447650253 | 0.00219956 |
| gadodiamide | C064925 | 2.748772101 | 0.00219956 |
| rottlerin | C085746 | 2.834668493 | 0.00219956 |
| alisol A 24-acetate | C457233 | 2.514101568 | 0.00219956 |
| Bleomycin | D001761 | 2.854868129 | 0.00219956 |
| Butylhydroxybutylnitrosamine | D002085 | 2.670168883 | 0.00219956 |
| G(M3) Ganglioside | D005679 | 2.602729152 | 0.00219956 |
| Thalidomide | D013792 | 2.856921494 | 0.00219956 |
| Nocodazole | D015739 | 2.863709986 | 0.00219956 |
| primycin | C009041 | 2.669201787 | 0.00239952 |
| cucurbitacin I | C038106 | 2.631296761 | 0.00239952 |
| N-(2-(4-bromocinnamylamino)ethyl)-5-isoquinolinesulfonamide | C063509 | 2.660875762 | 0.00239952 |
| fipronil | C082360 | 2.751127477 | 0.00239952 |
| fujiflavone P40 | C473894 | 2.62812933 | 0.00239952 |
| panduratin A | C482884 | 2.715144186 | 0.00239952 |
| Sorafenib | D000077157 | 2.917750305 | 0.00239952 |
| Alitretinoin | D000077556 | 2.912175647 | 0.00239952 |
| Fenbendazole | D005273 | 2.770941656 | 0.00239952 |
| Melatonin | D008550 | 2.969522564 | 0.00239952 |
| Nitric Oxide | D009569 | 2.680986814 | 0.00239952 |
| Toluene | D014050 | 2.866131967 | 0.00239952 |
| cinnamic aldehyde | C012843 | 2.776550318 | 0.00259948 |
| GW 4064 | C412815 | 2.792351731 | 0.00259948 |
| 4-fluorobenzoyl-TN-14003 | C477728 | 2.372679461 | 0.00259948 |
| Sterigmatocystin | D013241 | 2.89211117 | 0.00259948 |
| Triamcinolone | D014221 | 2.653164352 | 0.00279944 |
| Proton Pump Inhibitors | D054328 | 2.797441079 | 0.00279944 |
| beauvericin | C004456 | 2.761450848 | 0.0029994 |
| dibenzothiophene | C016366 | 2.639955353 | 0.0029994 |
| maduramicin | C018091 | 2.684087697 | 0.0029994 |
| thiodicarb | C027971 | 2.478252418 | 0.0029994 |
| 4-toluidine | C029370 | 2.768572839 | 0.0029994 |
| avobenzone | C049935 | 2.335635334 | 0.0029994 |
| perfluoro-n-undecanoic acid | C101814 | 2.750460432 | 0.0029994 |
| rofecoxib | C116926 | 2.616616205 | 0.0029994 |
| pyrazolanthrone | C432165 | 2.770900244 | 0.0029994 |
| 5-OH-BDE-47 | C580598 | 2.727594817 | 0.0029994 |
| Bromine | D001966 | 2.644955071 | 0.0029994 |
| Nitroglycerin | D005996 | 2.720811714 | 0.0029994 |
| mancozeb | C013099 | 2.726970665 | 0.00319936 |
| fumonisin B1 | C056933 | 2.726233216 | 0.00319936 |
| Prednisolone | D011239 | 2.725137752 | 0.00319936 |
| glycidol | C004312 | 2.713670157 | 0.00339932 |
| nickel subsulfide | C017557 | 2.687398297 | 0.00339932 |
| PD 123319 | C073402 | 2.588508642 | 0.00339932 |
| Fomepizole | D000077604 | 2.735658628 | 0.00339932 |
| Mevalonic Acid | D008798 | 2.78469748 | 0.00339932 |
| Sulfasalazine | D012460 | 2.697362339 | 0.00339932 |
| Silymarin | D012838 | 2.679368796 | 0.00339932 |
| Thiosemicarbazones | D013882 | 2.686834534 | 0.00339932 |
| dicyclanil | C121718 | 2.692845894 | 0.00359928 |
| Arecoline | D001115 | 2.634963079 | 0.00359928 |
| Bilirubin | D001663 | 2.748427375 | 0.00359928 |
| Camptothecin | D002166 | 2.708779443 | 0.00359928 |
| Palmitic Acid | D019308 | 2.568117647 | 0.00359928 |
| hexamethylene bisacetamide | C014026 | 2.579923496 | 0.00379924 |
| aristolochic acid II | C042310 | 2.608410655 | 0.00379924 |
| Imiquimod | D000077271 | 2.668633822 | 0.00379924 |
| Estrone | D004970 | 2.576345745 | 0.00379924 |
| Freund's Adjuvant | D005620 | 2.730692496 | 0.00379924 |
| Haloperidol | D006220 | 2.71999687 | 0.00379924 |
| Zymosan | D015054 | 2.620767873 | 0.00379924 |
| beta Carotene | D019207 | 2.589517488 | 0.00379924 |
| belinostat | C487081 | 2.712291218 | 0.0039992 |
| nordy | C510003 | 2.517610805 | 0.0039992 |
| Amsacrine | D000677 | 2.637095236 | 0.0039992 |
| Phosphoadenosine Phosphosulfate | D010724 | 2.334516231 | 0.0039992 |
| Terbutaline | D013726 | 2.722698387 | 0.0039992 |
| alachlor | C000188 | 2.693064211 | 0.00419916 |
| BHTOH-QM | C110172 | 2.513250192 | 0.00419916 |
| netoglitazone | C115174 | 2.369821073 | 0.00419916 |
| Quinolines | D011804 | 2.463244803 | 0.00419916 |
| Flavanones | D044950 | 2.5227874 | 0.00419916 |
| 10-(6'-ubiquinonyl)decyltriphenylphosphonium bromide | C476756 | 2.278165385 | 0.00439912 |
| LAQ824 | C477361 | 2.512561623 | 0.00439912 |
| Pesticides | D010575 | 2.528146634 | 0.00439912 |
| Scorpion Venoms | D012604 | 2.455078576 | 0.00439912 |
| Silicon Compounds | D017655 | 2.335234487 | 0.00439912 |
| tungsten carbide | C002802 | 2.916665411 | 0.00459908 |
| pifithrin | C121565 | 2.595707646 | 0.00459908 |
| Aluminum Oxide | D000537 | 2.552473891 | 0.00459908 |
| Fructose | D005632 | 2.509779744 | 0.00459908 |
| Hymecromone | D006923 | 2.579875505 | 0.00459908 |
| Zeranol | D015029 | 2.423406708 | 0.00459908 |
| (4-(7-chloroquinolin-4-yl)piperazino)(1-phenyl-5-(trifluoromethyl)-1H-pyrazol-4-yl)methanone | C000603489 | 2.205914103 | 0.00479904 |
| flumequine | C012976 | 2.520331952 | 0.00479904 |
| apicidin | C102351 | 2.65374045 | 0.00479904 |
| Uric Acid | D014527 | 2.490526994 | 0.00479904 |
| Luteolin | D047311 | 2.5171115 | 0.00479904 |
| gadolinium chloride | C038958 | 2.564162479 | 0.004999 |
| tetrakis(N-methyl-4-pyridiniumyl)porphine manganese(III) complex | C050950 | 2.46325866 | 0.004999 |
| tamibarotene | C061133 | 2.603185749 | 0.004999 |
| HS 1183 | C403900 | 2.341269951 | 0.004999 |
| Mirex | D008917 | 2.509849803 | 0.004999 |
| Plant Preparations | D028321 | 2.752630526 | 0.004999 |
| testosterone enanthate | C004648 | 2.558530373 | 0.00519896 |
| naringenin | C005273 | 2.492959035 | 0.00519896 |
| Anthocyanins | D000872 | 2.563802363 | 0.00519896 |
| Pentoxifylline | D010431 | 2.590495057 | 0.00519896 |
| Thimerosal | D013849 | 2.533138349 | 0.00519896 |
| pyrithione zinc | C010423 | 2.472356529 | 0.00539892 |
| phenanthrene | C031181 | 2.50938768 | 0.00539892 |
| Isoflavones | D007529 | 2.549388746 | 0.00539892 |
| Orphenadrine | D009966 | 2.518579565 | 0.00539892 |
| biochanin A | C004541 | 2.482159223 | 0.00559888 |
| uranyl acetate | C005460 | 2.436092265 | 0.00559888 |
| 4-dichlorobenzene | C018511 | 2.528581485 | 0.00559888 |
| pyrene | C030984 | 2.496802259 | 0.00559888 |
| Flame Retardants | D005411 | 2.459128197 | 0.00559888 |
| Melphalan | D008558 | 2.545440658 | 0.00559888 |
| 4-nitrophenyl acetate | C008642 | 2.470064667 | 0.00579884 |
| glyphosate | C010974 | 2.529952306 | 0.00579884 |
| HS-1199 | C503536 | 2.328662177 | 0.00579884 |
| Sunitinib | D000077210 | 2.474156504 | 0.00579884 |
| Dapsone | D003622 | 2.531276659 | 0.00579884 |
| Fenoldopam | D018818 | 2.42647939 | 0.00579884 |
| halofuginone | C010176 | 2.500270776 | 0.0059988 |
| cadmium sulfate | C037123 | 2.411956039 | 0.0059988 |
| CC-8490 | C495817 | 2.44623928 | 0.0059988 |
| 2-morpholin-4-yl-6-thianthren-1-yl-pyran-4-one | C495818 | 2.468179597 | 0.0059988 |
| Phosphatidylserines | D010718 | 2.275016625 | 0.0059988 |
| Retinoids | D012176 | 2.399320335 | 0.0059988 |
| HS 1200 | C479128 | 2.379312553 | 0.00619876 |
| Limonene | D000077222 | 2.59491603 | 0.00619876 |
| DDT | D003634 | 2.452866148 | 0.00619876 |
| Dicamba | D003996 | 2.261898774 | 0.00619876 |
| Levofloxacin | D064704 | 2.505272618 | 0.00619876 |
| bromobenzene | C032036 | 2.589350146 | 0.00639872 |
| 6-O-angeloylenolin | C531937 | 2.38988604 | 0.00639872 |
| Iodoacetates | D007461 | 2.380049788 | 0.00639872 |
| 4-(4-fluorophenyl)-2-(4-hydroxyphenyl)-5-(4-pyridyl)imidazole | C090942 | 2.58433007 | 0.00659868 |
| nutlin 3 | C482205 | 2.470979748 | 0.00659868 |
| AG-012986 | C512484 | 2.288640236 | 0.00659868 |
| Captan | D002215 | 2.359176748 | 0.00659868 |
| aspidin BB | C010302 | 2.307064784 | 0.00679864 |
| brassinolide | C023623 | 2.373876069 | 0.00679864 |
| 7-hydroxycoumarin | C031477 | 2.315998931 | 0.00679864 |
| vanadium pentoxide | C066075 | 2.325981942 | 0.00679864 |
| AMG 837 | C557933 | 2.44133701 | 0.00679864 |
| Boron Compounds | D001896 | 2.473609859 | 0.00679864 |
| Isoniazid | D007538 | 2.40781191 | 0.00679864 |
| Oxyquinoline | D015125 | 2.434530734 | 0.00679864 |
| Aphidicolin | D016590 | 2.334175949 | 0.00679864 |
| Xanthine | D019820 | 2.457763492 | 0.00679864 |
| chloroquine diphosphate | C023676 | 2.322265015 | 0.0069986 |
| Pioglitazone | D000077205 | 2.431459628 | 0.0069986 |
| Methylprednisolone Acetate | D000077555 | 2.308430427 | 0.0069986 |
| Sodium | D012964 | 2.481563257 | 0.0069986 |
| PD 156707 | C093321 | 2.131147582 | 0.00719856 |
| gambierol | C435770 | 2.255356535 | 0.00719856 |
| Ivermectin | D007559 | 2.378760676 | 0.00719856 |
| 1'-acetoxychavicol acetate | C047948 | 2.396053348 | 0.00739852 |
| hydroxytamoxifen | C475919 | 2.461240518 | 0.00739852 |
| Adenosine Diphosphate | D000244 | 2.35327927 | 0.00759848 |
| Oxytetracycline | D010118 | 2.236852507 | 0.00759848 |
| 8-Bromo Cyclic Adenosine Monophosphate | D015124 | 2.4319902 | 0.00759848 |
| Tocopherols | D024505 | 2.319121867 | 0.00759848 |
| daidzein | C004742 | 2.44256527 | 0.00779844 |
| sulfamic acid | C005741 | 2.133661689 | 0.00779844 |
| dibromoacetic acid | C088674 | 2.252635155 | 0.00779844 |
| abrine | C496492 | 2.416957415 | 0.00779844 |
| Catecholamines | D002395 | 2.264657161 | 0.00779844 |
| NADP | D009249 | 2.415426891 | 0.00779844 |
| Bisphenol A-Glycidyl Methacrylate | D017438 | 2.297510988 | 0.00779844 |
| N-nitrosodiethanolamine | C014129 | 2.372299078 | 0.0079984 |
| cardanol | C038590 | 2.230680868 | 0.0079984 |
| Ketone Bodies | D007657 | 2.393198334 | 0.0079984 |
| Nitrates | D009566 | 2.292176597 | 0.0079984 |
| Putrescine | D011700 | 2.368460624 | 0.0079984 |
| Benzophenanthridines | D053119 | 2.33063085 | 0.0079984 |
| sodium sulfite | C025026 | 2.237520372 | 0.00819836 |
| Pemetrexed | D000068437 | 2.291206266 | 0.00819836 |
| Acrolein | D000171 | 2.476581148 | 0.00819836 |
| Gallic Acid | D005707 | 2.326211015 | 0.00819836 |
| Vitamin D | D014807 | 2.360604919 | 0.00819836 |
| Astemizole | D016589 | 2.369252273 | 0.00819836 |
| Sevoflurane | D000077149 | 2.378418494 | 0.00839832 |
| Guaiacol | D006139 | 2.277907746 | 0.00839832 |
| Homocysteine | D006710 | 2.35407011 | 0.00839832 |
| hexyl cinnamic aldehyde | C080043 | 2.34894349 | 0.00859828 |
| prostaglandin A1 | C100573 | 2.447434748 | 0.00859828 |
| 2-phenyl-4-(3-pyridin-2-yl-1H-pyrazol-4-yl)pyridine | C502971 | 2.07026308 | 0.00859828 |
| Puromycin Aminonucleoside | D011692 | 2.362250435 | 0.00859828 |
| Amlodipine | D017311 | 2.387960971 | 0.00859828 |
| tungstate | C045951 | 2.220732746 | 0.00879824 |
| Atorvastatin | D000069059 | 2.2701732 | 0.00879824 |
| Nitroprusside | D009599 | 2.388339451 | 0.00879824 |
| danthron | C004315 | 2.357388413 | 0.0089982 |
| MK-886 | C060893 | 2.435872113 | 0.0089982 |
| IDN 5109 | C404743 | 2.134294881 | 0.0089982 |
| Metaproterenol | D009921 | 2.251763132 | 0.0089982 |
| cladosporin | C000488 | 2.324457802 | 0.00919816 |
| cinobufagin | C002471 | 2.179433176 | 0.00919816 |
| methoxyacetic acid | C013598 | 2.374063918 | 0.00919816 |
| deoxypodophyllotoxin | C014451 | 2.07236656 | 0.00919816 |
| tetracycline CMT-3 | C117155 | 2.294101053 | 0.00919816 |
| ircinin-1 | C506002 | 2.300751024 | 0.00919816 |
| hydrazine | C029424 | 2.363961644 | 0.00939812 |
| ursolic acid | C005466 | 2.347874696 | 0.00959808 |
| 4-vinyl-1-cyclohexene dioxide | C012606 | 2.410110378 | 0.00959808 |
| xanthatin | C022186 | 2.194667855 | 0.00959808 |
| vanadyl sulfate | C034028 | 2.301441381 | 0.00979804 |
| allyl sulfide | C038491 | 2.387600769 | 0.00979804 |
| kenpaullone | C119620 | 2.298186926 | 0.00979804 |
| Bucladesine | D003994 | 2.379653784 | 0.00979804 |
| Phloretin | D010693 | 2.232400586 | 0.00979804 |
| Quartz | D011791 | 2.255964144 | 0.00979804 |
| Fungal Polysaccharides | D062610 | 2.21729627 | 0.00979804 |
| fenvalerate | C017690 | 2.255338363 | 0.009998 |
| triclopyr | C032742 | 2.097733261 | 0.009998 |
| Desoxycorticosterone | D003900 | 2.245763335 | 0.009998 |
| Ethylenethiourea | D005031 | 2.277081866 | 0.009998 |
| Picloram | D010846 | 2.097733261 | 0.009998 |
| stattic | C517409 | 2.242725853 | 0.01019796 |
| Anisomycin | D000841 | 2.203325247 | 0.01019796 |
| Linoleic Acid | D019787 | 2.239156127 | 0.01019796 |
| lauryl gallate | C008259 | 2.138546677 | 0.01039792 |
| Methylprednisolone | D008775 | 2.351119533 | 0.01039792 |
| 3-phenoxybenzoic acid | C017618 | 2.062093777 | 0.01059788 |
| perfluorohexanesulfonic acid | C471071 | 2.311464807 | 0.01059788 |
| Estradiol | D004958 | 2.257445733 | 0.01059788 |
| pycnidione | C572726 | 2.056883921 | 0.01079784 |
| Sulfhydryl Compounds | D013438 | 2.048125582 | 0.01079784 |
| Isoxazoles | D007555 | 2.146762957 | 0.0109978 |
| geranylgeranyl pyrophosphate | C002963 | 2.256909347 | 0.01119776 |
| mofezolac | C054999 | 2.034994685 | 0.01119776 |
| TAK-875 | C557331 | 2.252327536 | 0.01119776 |
| Theophylline | D013806 | 2.32595 | 0.01119776 |
| 4-chlorobiphenyl | C006983 | 2.238500635 | 0.01139772 |
| Busulfan | D002066 | 2.158330503 | 0.01139772 |
| Hexamethonium | D018738 | 2.233869486 | 0.01139772 |
| butylbenzyl phthalate | C027561 | 2.291985098 | 0.01159768 |
| poly(propyleneimine) | C443641 | 2.236350195 | 0.01159768 |
| Graphite | D006108 | 2.260228206 | 0.01159768 |
| Polyethyleneimine | D011094 | 2.197126114 | 0.01159768 |
| ciprofibrate | C019304 | 2.240008528 | 0.01179764 |
| zomepirac | C020549 | 2.185815256 | 0.01179764 |
| cyclo(Trp-Asp-Pro-Val-Leu) | C072247 | 2.258768204 | 0.01179764 |
| N-((3S)-1-azabicyclo(2.2.2)oct-3-yl)-1H-indazole-3-carboxamide hydrochloride | C554954 | 2.244601616 | 0.01179764 |
| Biguanides | D001645 | 2.063622804 | 0.01179764 |
| Cholecalciferol | D002762 | 2.265452361 | 0.01179764 |
| vanillin | C100058 | 2.206227278 | 0.0119976 |
| Spiro Compounds | D013141 | 2.169552749 | 0.0119976 |
| Stavudine | D018119 | 2.170406915 | 0.01219756 |
| methyleugenol | C005223 | 2.216982202 | 0.01239752 |
| Chloroform | D002725 | 2.235661324 | 0.01239752 |
| Clozapine | D003024 | 2.209828405 | 0.01239752 |
| methyl antcinate A | C553655 | 2.113169444 | 0.012597481 |
| nodularin | C063998 | 2.147670992 | 0.012797441 |
| FR 139317 | C079574 | 2.041155531 | 0.012797441 |
| retene | C447880 | 2.139802336 | 0.012797441 |
| bicyclol | C477843 | 2.135684937 | 0.012997401 |
| Benomyl | D001542 | 2.180835274 | 0.012997401 |
| Chlorambucil | D002699 | 2.125074539 | 0.012997401 |
| Dexrazoxane | D064730 | 2.198420692 | 0.012997401 |
| tinuvin | C016151 | 2.274268997 | 0.013197361 |
| 1-(2-trifluoromethoxyphenyl)-2-nitroethanone | C576882 | 2.208451883 | 0.013197361 |
| Olive Oil | D000069463 | 2.233995099 | 0.013197361 |
| Phosphorylcholine | D010767 | 1.996186812 | 0.013197361 |
| cypermethrin | C017160 | 2.225539337 | 0.013397321 |
| bisperoxovanadium | C472777 | 2.10690086 | 0.013397321 |
| Uranium | D014501 | 2.25523671 | 0.013397321 |
| Polyphenols | D059808 | 2.171735833 | 0.013397321 |
| Clofarabine | D000077866 | 2.103500497 | 0.013597281 |
| Ampicillin | D000667 | 2.139080492 | 0.013597281 |
| Propoxur | D001074 | 1.978337587 | 0.013597281 |
| Etodolac | D017308 | 2.117204044 | 0.013597281 |
| hexylglutathione | C033550 | 1.91791879 | 0.013797241 |
| 4-tert-octylphenol | C105260 | 2.261813436 | 0.013797241 |
| Canertinib | C420268 | 2.032573281 | 0.013797241 |
| Roscovitine | D000077546 | 2.152640322 | 0.013797241 |
| Clotrimazole | D003022 | 2.144877703 | 0.013797241 |
| evodiamine | C049639 | 2.145008824 | 0.013997201 |
| Tadalafil | D000068581 | 2.111687037 | 0.013997201 |
| Hydroxychloroquine | D006886 | 1.999838537 | 0.013997201 |
| 3-deazaadenosine | C018258 | 1.951403583 | 0.014197161 |
| locostatin | C529490 | 2.044808579 | 0.014197161 |
| didymin | C552234 | 2.044808579 | 0.014197161 |
| Carnitine | D002331 | 2.180650696 | 0.014197161 |
| cumene hydroperoxide | C007164 | 2.201290274 | 0.014397121 |
| AC 93253 | C542662 | 2.12976293 | 0.014397121 |
| cyproconazole | C093628 | 2.224809348 | 0.014597081 |
| FR 173657 | C105311 | 1.966206617 | 0.014797041 |
| oligofectamine | C484027 | 2.164094012 | 0.014797041 |
| liposomal doxorubicin | C506643 | 2.120748318 | 0.014797041 |
| Minocycline | D008911 | 2.137200422 | 0.014797041 |
| Permethrin | D026023 | 2.175139373 | 0.014797041 |
| SB 225002 | C112019 | 1.93146045 | 0.014997001 |
| 6-(3-cyclopentyl-2-(4-(trifluoromethyl)-1H-imidazol-1-yl)propanamido)nicotinic acid | C571532 | 2.04602275 | 0.014997001 |
| Carotenoids | D002338 | 1.94672589 | 0.014997001 |
| Warfarin | D014859 | 2.094789671 | 0.015396921 |
| mibolerone | C100075 | 2.103939641 | 0.015596881 |
| Hemin | D006427 | 2.167096871 | 0.015796841 |
| Iron Compounds | D058085 | 2.100443329 | 0.015996801 |
| benzo(e)pyrene | C026487 | 2.17014614 | 0.016396721 |
| Plant Oils | D010938 | 2.133319434 | 0.016396721 |
| Spironolactone | D013148 | 2.176902316 | 0.016596681 |
| chlorendic acid | C035377 | 2.092297405 | 0.016796641 |
| CGP 42112A | C060894 | 1.913707543 | 0.016796641 |
| Aldosterone | D000450 | 2.061006979 | 0.016796641 |
| Protons | D011522 | 2.086310915 | 0.016796641 |
| tolmetin glucuronide | C056583 | 2.06358007 | 0.016996601 |
| indioside D | C428264 | 2.029731237 | 0.016996601 |
| Phthalic Acids | D010795 | 2.093402294 | 0.016996601 |
| UAB 30 | C112106 | 2.10893391 | 0.017196561 |
| Trimetazidine | D014292 | 1.983132047 | 0.017196561 |
| methylformamide | C002950 | 2.056238393 | 0.017396521 |
| bathocuproine sulfonate | C028559 | 2.115563984 | 0.017396521 |
| GCS-100 | C505531 | 1.902621668 | 0.017396521 |
| Polyamines | D011073 | 1.986352903 | 0.017396521 |
| Coal Ash | D060729 | 2.164682527 | 0.017396521 |
| beta-hexachlorocyclohexane | C023888 | 2.029068745 | 0.017596481 |
| AICA ribonucleotide | C031143 | 2.060285745 | 0.017596481 |
| Calcium | D002118 | 2.095936245 | 0.017596481 |
| Humic Substances | D006812 | 2.075517983 | 0.017596481 |
| Oxotremorine | D010095 | 2.006667867 | 0.017996401 |
| Gemfibrozil | D015248 | 2.079749084 | 0.017996401 |
| 2-dichlorobenzene | C004726 | 1.971135421 | 0.018196361 |
| methyl isocyanate | C008461 | 2.000230243 | 0.018196361 |
| retinol acetate | C009166 | 2.161441026 | 0.018196361 |
| manganese(III)-tetrakis(4-benzoic acid)porphyrin | C097284 | 2.122445337 | 0.018196361 |
| cucurbitacin E | C102326 | 2.041382469 | 0.018196361 |
| Tubocurarine | D014403 | 2.081030664 | 0.018196361 |
| Amino Acids | D000596 | 2.053157193 | 0.018396321 |
| Chondroitin Sulfates | D002809 | 2.058441573 | 0.018396321 |
| Clenbuterol | D002976 | 2.110967064 | 0.018396321 |
| Vitamin K 2 | D024482 | 2.033518745 | 0.018396321 |
| Mechlorethamine | D008466 | 2.080469585 | 0.018596281 |
| Adenosine-5'-(N-ethylcarboxamide) | D019830 | 2.003336617 | 0.018596281 |
| boldenone undecylenate | C001371 | 1.857815777 | 0.018796241 |
| parthenolide | C002669 | 2.001312072 | 0.018796241 |
| ethylene dichloride | C024565 | 2.032830105 | 0.018796241 |
| oxazolinodaunorubicin | C552952 | 1.961784355 | 0.018796241 |
| Monocrotaline | D016686 | 2.096993146 | 0.018796241 |
| Methazolamide | D008704 | 1.918652816 | 0.018996201 |
| linalool | C018584 | 2.0553436 | 0.019196161 |
| Am 580 | C068073 | 2.077238482 | 0.019196161 |
| Bacitracin | D001414 | 2.029280996 | 0.019196161 |
| Boron | D001895 | 1.820473988 | 0.019196161 |
| Carmustine | D002330 | 2.089804535 | 0.019196161 |
| Fusaric Acid | D005669 | 1.950280808 | 0.019196161 |
| Benzyl Alcohol | D019905 | 1.91177337 | 0.019196161 |
| hexabromocyclododecane | C089796 | 2.055754684 | 0.019396121 |
| macrophage stimulatory lipopeptide 2 | C106335 | 2.038283832 | 0.019396121 |
| Sulfamethoxazole | D013420 | 2.010487048 | 0.019396121 |
| hydroperoxyisophosphamide | C012353 | 1.93539569 | 0.019596081 |
| ferric nitrilotriacetate | C020326 | 2.020448571 | 0.019596081 |
| propionic acid | C029658 | 1.934120226 | 0.019596081 |
| polydatin | C058229 | 2.006422707 | 0.019596081 |
| pirfenidone | C093844 | 2.007653937 | 0.019596081 |
| tetraarsenic tetrasulfide | C534735 | 2.00183574 | 0.019596081 |
| 1-Deoxynojirimycin | D017485 | 1.998051316 | 0.019596081 |
| BP-1-102 | C000595192 | 1.946965208 | 0.019796041 |
| Sulfapyridine | D013427 | 1.978287311 | 0.019796041 |
| Telmisartan | D000077333 | 1.98353185 | 0.019996001 |
| Brefeldin A | D020126 | 1.994192043 | 0.019996001 |
| withaferin A | C009684 | 1.997979969 | 0.020195961 |
| taurine-ursodeoxycholate conjugate | C030141 | 1.998600901 | 0.020195961 |
| anacardic acid | C088115 | 2.039970253 | 0.020195961 |
| adefovir dipivoxil | C106812 | 2.013154629 | 0.020195961 |
| Niacin | D009525 | 2.091323896 | 0.020195961 |
| Carboplatin | D016190 | 2.02226584 | 0.020195961 |
| licochalcone B | C541528 | 1.957194979 | 0.020395921 |
| Berberine | D001599 | 1.996929549 | 0.020395921 |
| stannous chloride | C023599 | 1.958210516 | 0.020595881 |
| Grape Seed Proanthocyanidins | C511402 | 2.03733033 | 0.020595881 |
| Temozolomide | D000077204 | 2.062395105 | 0.020595881 |
| Chrysenes | D002911 | 1.969988902 | 0.020595881 |
| Losartan | D019808 | 1.98550746 | 0.020595881 |
| Vanadium | D014639 | 2.039808509 | 0.020795841 |
| 8-bromocyclic GMP | C016276 | 2.004399505 | 0.020995801 |
| benzo(b)fluorene | C041512 | 1.971338596 | 0.020995801 |
| xanthohumol | C104536 | 2.057296573 | 0.021395721 |
| magnolol | C005498 | 1.944268569 | 0.021595681 |
| coptisine | C034384 | 1.896584003 | 0.021595681 |
| Betamethasone | D001623 | 1.961200104 | 0.021595681 |
| 4-Chloromercuribenzenesulfonate | D002731 | 1.946625477 | 0.021595681 |
| Pentosan Sulfuric Polyester | D010426 | 1.995533485 | 0.021595681 |
| Platinum | D010984 | 1.986469177 | 0.021595681 |
| navitoclax | C528561 | 1.930024881 | 0.021995601 |
| Chlorophyll | D002734 | 1.940604915 | 0.021995601 |
| Toxaphene | D014112 | 2.013107999 | 0.021995601 |
| momfluorothrin | C000627226 | 2.03332474 | 0.022195561 |
| Ethidium | D004996 | 1.963698746 | 0.022195561 |
| 4-nonylphenol | C041594 | 2.039221095 | 0.022395521 |
| importazole | C568452 | 1.899256238 | 0.022395521 |
| Anthraquinones | D000880 | 1.954124833 | 0.022395521 |
| ferrous sulfate | C020748 | 2.034431921 | 0.022595481 |
| corosolic acid | C113861 | 1.947400769 | 0.022595481 |
| Saponins | D012503 | 1.964512762 | 0.022595481 |
| CGS 27023A | C097658 | 1.859334664 | 0.022795441 |
| maslinic acid | C412811 | 1.978082733 | 0.022995401 |
| Chenodeoxycholic Acid | D002635 | 2.000627618 | 0.022995401 |
| Pyrimidines | D011743 | 1.957881682 | 0.022995401 |
| Tolbutamide | D014044 | 1.90600533 | 0.022995401 |
| methyl salicylate | C033069 | 1.960257065 | 0.023195361 |
| etomoxir | C054207 | 1.908468384 | 0.023195361 |
| archazolid B | C519728 | 1.996463355 | 0.023195361 |
| Chlorine | D002713 | 2.012315704 | 0.023195361 |
| morin | C008548 | 1.934451105 | 0.023395321 |
| nimbolide | C042198 | 1.940378174 | 0.023395321 |
| 7-hydroxystaurosporine | C054852 | 1.947334804 | 0.023395321 |
| bleomycetin | C025703 | 1.985114441 | 0.023595281 |
| resmethrin | C100036 | 1.860164626 | 0.023595281 |
| Farnesol | D005204 | 1.968718933 | 0.023795241 |
| borrelidin | C005066 | 1.836934899 | 0.023995201 |
| BL 11282 | C423844 | 1.932467794 | 0.023995201 |
| di-(2-ethylhexyl) terephthalate | C053316 | 1.832568173 | 0.024195161 |
| curdlan | C038459 | 1.897692148 | 0.024395121 |
| cafestol | C053400 | 1.883714431 | 0.024395121 |
| 6-isopropoxy-9-oxoxanthene-2-carboxylic acid | C053876 | 1.964422083 | 0.024395121 |
| Megestrol Acetate | D019290 | 1.897355949 | 0.024395121 |
| Glucuronides | D020719 | 1.867663383 | 0.024395121 |
| bismuth tripotassium dicitrate | C002791 | 1.975564449 | 0.024595081 |
| fenofibric acid | C006012 | 1.930799106 | 0.024595081 |
| Vinyl Chloride | D014752 | 1.96256098 | 0.024595081 |
| allose | C002055 | 1.776740152 | 0.024795041 |
| Roxarsone | D012406 | 1.861791996 | 0.024795041 |
| Thiotepa | D013852 | 1.938013284 | 0.024795041 |
| 3-hydroxybenzo(a)pyrene | C021338 | 1.857204706 | 0.024995001 |
| Albuterol | D000420 | 2.000014165 | 0.024995001 |
| farnesylthiosalicylic acid | C093323 | 1.883266393 | 0.025194961 |
| Amoxicillin | D000658 | 1.800792339 | 0.025194961 |
| Colforsin | D005576 | 1.958830336 | 0.025194961 |
| Thioctic Acid | D008063 | 1.893814859 | 0.025194961 |
| Vigabatrin | D020888 | 1.935291634 | 0.025394921 |
| N-caproylsphingosine | C101954 | 1.874074226 | 0.025594881 |
| Alloxan | D000496 | 1.931957355 | 0.025594881 |
| Saccharin | D012439 | 1.921644603 | 0.025594881 |
| estragole | C007633 | 1.893094248 | 0.025794841 |
| beta-glycerophosphoric acid | C031463 | 1.888101161 | 0.025794841 |
| Pyrogallol | D011748 | 2.010168634 | 0.025794841 |
| Oxidants | D016877 | 1.834790943 | 0.025794841 |
| chromic oxide | C023600 | 1.889020357 | 0.025994801 |
| Heparin | D006493 | 1.920377644 | 0.025994801 |
| Dextran Sulfate | D016264 | 1.980762747 | 0.025994801 |
| testosterone undecanoate | C010792 | 1.917174022 | 0.026194761 |
| mizoribine | C010052 | 1.777578646 | 0.026394721 |
| Glu-P-2 | C023847 | 1.84290224 | 0.026394721 |
| Proteoglycans | D011509 | 1.832869249 | 0.026394721 |
| Prostaglandin D2 | D015230 | 1.901555153 | 0.026394721 |
| RTKI cpd | C101044 | 1.980940512 | 0.026594681 |
| CI 1044 | C418118 | 1.957644698 | 0.026794641 |
| lonafarnib | C115354 | 1.877195701 | 0.026994601 |
| Propolis | D011429 | 1.92843285 | 0.026994601 |
| nobiletin | C008661 | 1.877838922 | 0.027394521 |
| 2'-cyano-2'-deoxyarabinofuranosylcytosine | C070279 | 1.768079551 | 0.027394521 |
| catechol | C034221 | 1.907509333 | 0.027594481 |
| cariporide | C093373 | 1.83709694 | 0.027594481 |
| Cannabidiol | D002185 | 1.919134603 | 0.027594481 |
| Chlorzoxazone | D002753 | 1.807243224 | 0.027594481 |
| Iron-Dextran Complex | D007505 | 1.887000999 | 0.027594481 |
| LL-202 | C000589248 | 1.838122983 | 0.027794441 |
| HT-2 toxin | C012351 | 1.89830536 | 0.027794441 |
| metolachlor | C051786 | 1.861749681 | 0.027794441 |
| Margarine | D008383 | 1.875430455 | 0.027794441 |
| pimagedine | C004479 | 1.962560317 | 0.027994401 |
| propamocarb | C033205 | 1.862719778 | 0.028194361 |
| 17-(dimethylaminoethylamino)-17-demethoxygeldanamycin | C448659 | 1.875885779 | 0.028194361 |
| NT020 formula | C522862 | 1.864371979 | 0.028194361 |
| Gefitinib | D000077156 | 1.854127053 | 0.028394321 |
| Hydrocortisone | D006854 | 1.916506868 | 0.028394321 |
| pluronic block copolymer p85 | C422648 | 1.84952844 | 0.028594281 |
| N-(2-(3H-indol-3-yl)ethyl)-9-isopropyl-2-(5-methyl-3-pyridyl)purin-6-amine | C560625 | 1.845487632 | 0.028594281 |
| puag-haad | C034912 | 1.893179697 | 0.028794241 |
| molybdate | C044659 | 1.82126101 | 0.028794241 |
| ethylene dimethanesulfonate | C002994 | 1.884178362 | 0.028994201 |
| methyldithiocarbamate | C008435 | 1.91031092 | 0.028994201 |
| 2-tert-butylhydroquinone | C018855 | 1.826895529 | 0.028994201 |
| caffeic acid phenethyl ester | C055494 | 1.937526369 | 0.028994201 |
| Lactic Acid | D019344 | 1.898244041 | 0.029194161 |
| UMI-77 | C000592878 | 1.796139788 | 0.029394121 |
| Perfume | D010476 | 1.900908928 | 0.029394121 |
| Ribavirin | D012254 | 1.894005908 | 0.029594081 |
| Sodium Dodecyl Sulfate | D012967 | 1.842698165 | 0.029594081 |
| apocarotenal | C004984 | 1.831271856 | 0.029794041 |
| N-acetylsphingosine | C064769 | 1.845878071 | 0.029794041 |
| Allylamine | D000499 | 1.900779571 | 0.029794041 |
| GKT137831 | C576694 | 1.766688542 | 0.029994001 |
| Fatty Acids | D005227 | 1.858513609 | 0.029994001 |
| o-Phthalaldehyde | D009764 | 1.834321883 | 0.029994001 |
| genipin | C007834 | 1.92844721 | 0.030193961 |
| fosbretabulin | C058728 | 1.808153291 | 0.030193961 |
| acetylbritannilatone | C487680 | 1.826398453 | 0.030193961 |
| KCC 009 | C510621 | 1.772020648 | 0.030193961 |
| cyanobacterial toxin | C014986 | 1.789460469 | 0.030393921 |
| Ro 31-8220 | C064758 | 1.826322111 | 0.030393921 |
| methyl jasmonate | C072239 | 1.825753789 | 0.030393921 |
| 4-nitroaniline | C019498 | 1.797207567 | 0.030593881 |
| Mitotane | D008939 | 1.884451824 | 0.030593881 |
| glucosamine 3-O-sulfate | C065632 | 1.720345365 | 0.030993801 |
| 4-(2-aminoethyl)benzenesulfonylfluoride | C002010 | 1.803815222 | 0.031193761 |
| gingerol | C007845 | 1.808853942 | 0.031193761 |
| zomepirac glucuronide | C027368 | 1.849243057 | 0.031193761 |
| Oxaloacetic Acid | D062907 | 1.753456812 | 0.031193761 |
| n-tetradecane | C024713 | 1.87064235 | 0.031393721 |
| Ketoconazole | D007654 | 1.880806239 | 0.031393721 |
| Titanium | D014025 | 1.818688373 | 0.031393721 |
| Trientine | D014266 | 1.8203861 | 0.031393721 |
| Pertussis Toxin | D037342 | 1.883684795 | 0.031393721 |
| catalase-polyethylene glycol | C070295 | 1.814216003 | 0.031593681 |
| VX680 | C484810 | 1.764307139 | 0.031793641 |
| Acetyl Coenzyme A | D000105 | 1.768768893 | 0.031793641 |
| mono-(2-ethylhexyl)phthalate | C016599 | 1.847181364 | 0.031993601 |
| Hydroxyapatites | D006882 | 1.754744968 | 0.031993601 |
| alpha-naphthoflavone | C011512 | 1.836446774 | 0.032393521 |
| Galactosamine | D005688 | 1.818379711 | 0.032593481 |
| Ketorolac | D020910 | 1.828198136 | 0.032593481 |
| cumene | C015763 | 1.824733282 | 0.032793441 |
| Catechin | D002392 | 1.895132648 | 0.032793441 |
| Trypsin Inhibitors | D014361 | 1.755824266 | 0.032993401 |
| flusilazole | C061365 | 1.868854045 | 0.033193361 |
| tetraethylenepentamine | C034269 | 1.762162446 | 0.033393321 |
| chrysin | C043561 | 1.812324363 | 0.033393321 |
| Cholesterol | D002784 | 1.874751292 | 0.033393321 |
| Acids | D000143 | 1.776339192 | 0.033593281 |
| ferric ammonium citrate | C013531 | 1.812044774 | 0.033793241 |
| Lutein | D014975 | 1.832706793 | 0.033793241 |
| Isoflurane | D007530 | 1.825734405 | 0.033993201 |
| Gliclazide | D005907 | 1.745566215 | 0.034193161 |
| Lipids | D008055 | 1.785296811 | 0.034193161 |
| 2-hydroxymethyl-2-methoxymethylazabicyclo(2.2.2)octan-3-one | C533410 | 1.847301274 | 0.034393121 |
| dibutyldichlorotin | C020735 | 1.791379172 | 0.034593081 |
| phenylhydrazine | C030299 | 1.788712124 | 0.034593081 |
| Hesperidin | D006569 | 1.761015853 | 0.034593081 |
| Hexachlorophene | D006582 | 1.752732009 | 0.034593081 |
| diazoxon | C000912 | 1.721371764 | 0.034793041 |
| cinnamyl alcohol | C020722 | 1.787996442 | 0.034793041 |
| Lapatinib | D000077341 | 1.789851011 | 0.034793041 |
| Mebendazole | D008463 | 1.811308452 | 0.034793041 |
| Milrinone | D020105 | 1.786828175 | 0.034793041 |
| Matrix Metalloproteinase Inhibitors | D061965 | 1.72969966 | 0.034793041 |
| spautin-1 | C000591235 | 1.664925068 | 0.034993001 |
| oltipraz | C026209 | 1.798831852 | 0.034993001 |
| benzyl bromide | C038682 | 1.698310981 | 0.034993001 |
| Lycopene | D000077276 | 1.822717145 | 0.034993001 |
| Orlistat | D000077403 | 1.776258143 | 0.034993001 |
| lauric acid | C030358 | 1.719825533 | 0.035192961 |
| Calcium Chloride | D002122 | 1.77714571 | 0.035192961 |
| Tungsten | D014414 | 1.817563447 | 0.035192961 |
| Chlormethiazole | D002719 | 1.778127505 | 0.035592881 |
| Cytochalasin B | D003571 | 1.768930657 | 0.035592881 |
| Proadifen | D011335 | 1.778296515 | 0.035592881 |
| 15-acetyldeoxynivalenol | C046760 | 1.787978345 | 0.035792841 |
| Phosphatidylcholines | D010713 | 1.806187424 | 0.035992801 |
| triphenyl phosphate | C005445 | 1.759049009 | 0.036192761 |
| Mycotoxins | D009183 | 1.75967075 | 0.036192761 |
| romidepsin | C087123 | 1.756066832 | 0.036392721 |
| Hexachlorobenzene | D006581 | 1.768615437 | 0.036392721 |
| S-Adenosylhomocysteine | D012435 | 1.73314255 | 0.036392721 |
| 1-aminobenzotriazole | C033020 | 1.765427484 | 0.036592681 |
| Halogenated Diphenyl Ethers | D055768 | 1.754044669 | 0.036592681 |
| TO-901317 | C423915 | 1.761369675 | 0.036992601 |
| EUK-189 | C436526 | 1.70592047 | 0.036992601 |
| artemisic acid | C047721 | 1.675142398 | 0.037192561 |
| WZ4003 | C586755 | 1.69550105 | 0.037192561 |
| garcinol | C054597 | 1.769459962 | 0.037392521 |
| perfluoro-n-heptanoic acid | C101815 | 1.746013664 | 0.037392521 |
| Adenosine | D000241 | 1.741454092 | 0.037392521 |
| BO-1012 | C000591165 | 1.63919525 | 0.037592482 |
| Zinc Sulfate | D019287 | 1.776822741 | 0.037592482 |
| beryllium sulfate | C020711 | 1.780448106 | 0.037992402 |
| fenbuconazole | C504383 | 1.743677211 | 0.037992402 |
| selenomethylselenocysteine | C002979 | 1.749223632 | 0.038192362 |
| hesperetin | C013015 | 1.782314059 | 0.038192362 |
| indopan | C015293 | 1.671037241 | 0.038192362 |
| Cetirizine | D017332 | 1.696880949 | 0.038192362 |
| Sucrose | D013395 | 1.752293599 | 0.038392322 |
| 2-chloro-5-nitrobenzanilide | C457499 | 1.775649839 | 0.038592282 |
| brassinin | C089020 | 1.649017388 | 0.038792242 |
| Acarbose | D020909 | 1.740495288 | 0.038992202 |
| TEMPO | C003959 | 1.687330802 | 0.039192162 |
| fucoidan | C007789 | 1.712947661 | 0.039192162 |
| quinoline | C037219 | 1.748965874 | 0.039192162 |
| Trabectedin | D000077606 | 1.683323166 | 0.039192162 |
| bis(maltolato)oxovanadium(IV) | C074212 | 1.727618749 | 0.039392122 |
| vinyl carbamate | C017963 | 1.753240565 | 0.039592082 |
| Protein Kinase Inhibitors | D047428 | 1.754209018 | 0.039592082 |
| phenoxyacetic acid | C415326 | 1.707846892 | 0.039792042 |
| MT19c compound | C568376 | 1.767496888 | 0.039792042 |
| Furosemide | D005665 | 1.721204099 | 0.039792042 |
| 5-(4-ethylpiperazin-1-ylmethyl)pyridin-2-yl)-(5-fluoro-4-(7-fluoro-3-isopropyl-2-methyl-3H-benzimidazol-5-yl)pyrimidin-2-yl)amine | C000590451 | 1.688370568 | 0.039992002 |
| oxybenzone | C005290 | 1.768173745 | 0.039992002 |
| perillyl alcohol | C032208 | 1.678131895 | 0.039992002 |
| beryllium fluoride | C035982 | 1.648452913 | 0.039992002 |
| Valsartan | D000068756 | 1.784496463 | 0.039992002 |
| Ovalbumin | D010047 | 1.756282157 | 0.040191962 |
| 4-hydroxyestrone | C026418 | 1.687336105 | 0.040391922 |
| Carbamates | D002219 | 1.717363863 | 0.040591882 |
| AZD 6244 | C517975 | 1.759724564 | 0.040791842 |
| Simazine | D012839 | 1.722808315 | 0.040791842 |
| nano-diamino-tetrac | C000626766 | 1.662739407 | 0.040991802 |
| Dehydroascorbic Acid | D003683 | 1.711799503 | 0.040991802 |
| M-VAC protocol | C044361 | 1.756545611 | 0.041191762 |
| gamma-Linolenic Acid | D017965 | 1.717022091 | 0.041191762 |
| N-phenyl-7-(hydroxyimino)cyclopropa(b)chromen-1a-carboxamide | C442478 | 1.653083704 | 0.041791642 |
| fenamidone | C540355 | 1.75315923 | 0.041791642 |
| Verteporfin | D000077362 | 1.642131619 | 0.041791642 |
| Arachidonic Acid | D016718 | 1.758348183 | 0.041991602 |
| 3-hydroxy-4-prenyl-5-methoxystilbene-2-carboxylic acid | C549543 | 1.666720167 | 0.042191562 |
| farglitazar | C422415 | 1.680930008 | 0.042391522 |
| citral | C007076 | 1.667401408 | 0.042591482 |
| cyhalothrin | C037304 | 1.731320975 | 0.042791442 |
| 3-nitrobenzanthrone | C117220 | 1.68572874 | 0.042791442 |
| 4-oxoretinoic acid | C002202 | 1.74753667 | 0.042991402 |
| 1-hydroxypyrene | C033146 | 1.686719214 | 0.042991402 |
| Formoterol Fumarate | D000068759 | 1.645572179 | 0.042991402 |
| andrographolide | C030419 | 1.721752895 | 0.043191362 |
| geniposide | C007835 | 1.678951651 | 0.043391322 |
| Acetic Acid | D019342 | 1.72059782 | 0.043391322 |
| piperine | C008922 | 1.721955118 | 0.043591282 |
| bazedoxifene | C447119 | 1.727644186 | 0.043591282 |
| manganese tetrakis-(N-ethyl-2 pyridyl) porphyrin | C520506 | 1.665694 | 0.043591282 |
| Quinic Acid | D011801 | 1.693080707 | 0.043591282 |
| ziyuglycoside II | C000588918 | 1.663414541 | 0.043791242 |
| Cysteine | D003545 | 1.68966897 | 0.043791242 |
| Galactose | D005690 | 1.732535627 | 0.043791242 |
| Lidocaine | D008012 | 1.682380815 | 0.043791242 |
| pelargonic acid | C008776 | 1.697200449 | 0.043991202 |
| Trichloroacetic Acid | D014238 | 1.682468099 | 0.043991202 |
| oleoyl-estrone | C108709 | 1.667591256 | 0.044191162 |
| Water | D014867 | 1.687924374 | 0.044191162 |
| 5-alpha-Dihydroprogesterone | D043582 | 1.662248725 | 0.044191162 |
| benzamil | C013407 | 1.654358289 | 0.044391122 |
| testosterone 17 beta-cypionate | C016131 | 1.614157424 | 0.044391122 |
| N-benzoyl-12-nitrodehydroabietylamine-7-one | C576077 | 1.622842708 | 0.044391122 |
| Monocrotophos | D008999 | 1.699638719 | 0.044391122 |
| abamectin | C048324 | 1.637276073 | 0.044591082 |
| azoxystrobin | C087670 | 1.675285801 | 0.044591082 |
| veliparib | C521013 | 1.648023741 | 0.044591082 |
| methylparaben | C015358 | 1.709035359 | 0.044791042 |
| Azides | D001386 | 1.646492726 | 0.044791042 |
| Fludrocortisone | D005438 | 1.598821764 | 0.044791042 |
| Phosphamidon | D010707 | 1.604061038 | 0.044791042 |
| 1-UFT protocol | C031903 | 1.665991042 | 0.044991002 |
| benzyloxycarbonyl-valyl-alanyl-aspartic acid | C517629 | 1.659627895 | 0.044991002 |
| Brimonidine Tartrate | D000068438 | 1.608680276 | 0.044991002 |
| nickel acetate | C119536 | 1.632441845 | 0.045190962 |
| 2-butenal | C012796 | 1.689187957 | 0.045390922 |
| norcantharidin | C069741 | 1.647055148 | 0.045390922 |
| 4-hydroxybenzoic acid | C038193 | 1.585970629 | 0.045590882 |
| candesartan | C081643 | 1.680456689 | 0.045590882 |
| tetrathiomolybdate | C020809 | 1.680732563 | 0.045790842 |
| 4-nitrophenol | C024836 | 1.687480846 | 0.045790842 |
| antrocin | C558369 | 1.630912062 | 0.045790842 |
| Ranitidine | D011899 | 1.653926622 | 0.045990802 |
| Surface-Active Agents | D013501 | 1.661743156 | 0.045990802 |
| Teniposide | D013713 | 1.638943906 | 0.046190762 |
| cicaprost | C043867 | 1.595360685 | 0.046390722 |
| ARV-825 | C000606252 | 1.587204996 | 0.046590682 |
| alpha-hexachlorocyclohexane | C040534 | 1.676928533 | 0.046590682 |
| Atrasentan | D000077868 | 1.647127373 | 0.046590682 |
| Amiloride | D000584 | 1.666228571 | 0.046590682 |
| Kainic Acid | D007608 | 1.694780034 | 0.046590682 |
| darinaparsin | C515055 | 1.68226416 | 0.046790642 |
| Peptides | D010455 | 1.671977127 | 0.046790642 |
| Porphyrins | D011166 | 1.645387367 | 0.046790642 |
| cobra venom factor | C016310 | 1.576549002 | 0.047190562 |
| GW8510 | C500810 | 1.629231014 | 0.047190562 |
| pirimicarb | C011994 | 1.60938045 | 0.047390522 |
| KN 62 | C063302 | 1.652484692 | 0.047390522 |
| kahweol | C053401 | 1.649632414 | 0.047590482 |
| candoxin | C459604 | 1.700505895 | 0.047790442 |
| bisindolylmaleimide I | C070515 | 1.624274757 | 0.047990402 |
| S-Adenosylmethionine | D012436 | 1.634471486 | 0.047990402 |
| gold (III) porphyrin 1a | C508665 | 1.678109531 | 0.048190362 |
| Soil Pollutants | D012989 | 1.654260527 | 0.048190362 |
| cobalt sulfate | C026305 | 1.642245928 | 0.048790242 |
| 2-(4-isobutylphenyl)propionylmethanesulfonamide | C490707 | 1.546848353 | 0.048790242 |
| PK 11195 | C037850 | 1.614599061 | 0.048990202 |
| 4'-cyanobiphenyl-4-sulfonic acid (6-aminopyridin-2-yl)amide | C525872 | 1.561406769 | 0.049190162 |
| silybin-phytosome | C518471 | 1.536336023 | 0.049390122 |
| GSK525762A | C554645 | 1.623611436 | 0.049590082 |
| Heme | D006418 | 1.647691778 | 0.049590082 |
| alloin | C006457 | 1.539416702 | 0.049790042 |
| Go 6976 | C081021 | 1.651792628 | 0.049790042 |
| decanoic acid | C031071 | 1.591665647 | 0.049990002 |
| grifolin | C058858 | 1.589798706 | 0.049990002 |

^1^ Chemical ID: The ID of the chemicals in [Comparative Toxicogenomics Database](http://ctdbase.org/)(CTD).

^2^ NES: Normalized enrichment score.

## Supplementary Table 4. 104 common chemicals identified for colon cancer after a comparative of GWAS and mRNA GSEA results. (P value＜0.05)

| Chemical Name | Chemical ID^1^ | NES1^2^ | P-value1^3^ | NES2^4^ | P-value2^5^ |
| --- | --- | --- | --- | --- | --- |
| 1-methylanthracene | C051246 | 2.21576877 | 0.014797 | 4.145546 | 0.0002 |
| 1-nitropyrene | C032668 | 1.93521812 | 0.023395 | 4.810161 | 0.0002 |
| Acrolein | D000171 | 2.16029941 | 0.015197 | 2.476581 | 0.008198 |
| Aerosols | D000336 | 3.12516923 | 0.0016 | 5.187294 | 0.0002 |
| Am 580 | C068073 | 2.87048393 | 0.0024 | 2.077238 | 0.019196 |
| Anisomycin | D000841 | 1.80259813 | 0.037193 | 2.203325 | 0.010198 |
| Antirheumatic Agents | D018501 | 3.5802048 | 0.0002 | 6.506737 | 0.0002 |
| Arachidonic Acid | D016718 | 2.70540841 | 0.003199 | 1.758348 | 0.041992 |
| archazolid B | C519728 | 2.00200278 | 0.019396 | 1.996463 | 0.023195 |
| aristolochic acid I | C000228 | 1.78169252 | 0.036393 | 2.846424 | 0.0016 |
| aristolochic acid II | C042310 | 2.31088736 | 0.009398 | 2.608411 | 0.003799 |
| aurapten | C105832 | 1.69823826 | 0.036593 | 2.984325 | 0.0008 |
| AZM551248 | C547126 | 1.88193377 | 0.029794 | 9.513285 | 0.0002 |
| bathocuproine sulfonate | C028559 | 1.73363237 | 0.044391 | 2.115564 | 0.017397 |
| Benzbromarone | D001553 | 1.93604115 | 0.028594 | 3.74286 | 0.0002 |
| bis(4-hydroxyphenyl)sulfone | C543008 | 2.03831319 | 0.023195 | 5.837208 | 0.0002 |
| Bromine | D001966 | 1.93440617 | 0.023795 | 2.644955 | 0.002999 |
| butylidenephthalide | C026105 | 2.57360322 | 0.004199 | 2.949074 | 0.001 |
| C646 compound | C584509 | 1.68461972 | 0.044391 | 12.64983 | 0.0002 |
| cafestol | C053400 | 1.72428924 | 0.040792 | 1.883714 | 0.024395 |
| Carboplatin | D016190 | 2.57221524 | 0.005199 | 2.022266 | 0.020196 |
| Catechin | D002392 | 2.61456161 | 0.005199 | 1.895133 | 0.032793 |
| Chenodeoxycholic Acid | D002635 | 1.976472 | 0.024395 | 2.000628 | 0.022995 |
| Chloroprene | D002737 | 2.015719 | 0.022396 | 4.354951 | 0.0002 |
| Cholecalciferol | D002762 | 1.67168352 | 0.04919 | 2.265452 | 0.011798 |
| Clofibric Acid | D002995 | 2.60384551 | 0.005199 | 7.741625 | 0.0002 |
| Cyclophosphamide | D003520 | 2.34180037 | 0.012198 | 4.96917 | 0.0002 |
| Cysteine | D003545 | 2.3590243 | 0.007798 | 1.689669 | 0.043791 |
| darinaparsin | C515055 | 1.79040442 | 0.035793 | 1.682264 | 0.046791 |
| Ethionine | D005001 | 2.11950427 | 0.018396 | 3.624434 | 0.0004 |
| ferric nitrilotriacetate | C020326 | 2.14903339 | 0.015997 | 2.020449 | 0.019596 |
| fludarabine | C024352 | 1.83665831 | 0.032993 | 4.09474 | 0.0002 |
| fluoranthene | C007738 | 1.81998982 | 0.038392 | 4.665718 | 0.0002 |
| Fluorouracil | D005472 | 1.66302559 | 0.04859 | 9.443149 | 0.0002 |
| Fulvestrant | D000077267 | 1.78309183 | 0.037393 | 6.426685 | 0.0002 |
| Fungal Polysaccharides | D062610 | 1.94218279 | 0.024395 | 2.217296 | 0.009798 |
| gold (III) porphyrin 1a | C508665 | 1.92238924 | 0.024995 | 1.67811 | 0.04819 |
| Graphite | D006108 | 2.13149168 | 0.016997 | 2.260228 | 0.011598 |
| hexamethylene bisacetamide | C014026 | 1.74329916 | 0.040992 | 2.579923 | 0.003799 |
| Hydrogen Peroxide | D006861 | 1.84532257 | 0.034993 | 13.63099 | 0.0002 |
| Imatinib Mesylate | D000068877 | 1.93358209 | 0.026795 | 2.89379 | 0.0014 |
| importazole | C568452 | 1.72782033 | 0.037792 | 1.899256 | 0.022396 |
| Isoflavones | D007529 | 1.71905512 | 0.045191 | 2.549389 | 0.005399 |
| Isoniazid | D007538 | 2.14757717 | 0.016397 | 2.407812 | 0.006799 |
| Ivermectin | D007559 | 1.9473634 | 0.029194 | 2.378761 | 0.007199 |
| Ketamine | D007649 | 1.7128947 | 0.045791 | 5.120745 | 0.0002 |
| LG 100815 | C533894 | 3.21317886 | 0.0004 | 3.348366 | 0.0004 |
| Lidocaine | D008012 | 2.00373212 | 0.025795 | 1.682381 | 0.043791 |
| Medroxyprogesterone Acetate | D017258 | 1.66999349 | 0.04779 | 4.220949 | 0.0004 |
| Mercuric Chloride | D008627 | 2.02449893 | 0.020596 | 6.040111 | 0.0002 |
| Methylnitronitrosoguanidine | D008769 | 1.79077245 | 0.039392 | 3.735168 | 0.0002 |
| Methyltestosterone | D008777 | 1.87285561 | 0.031394 | 7.015748 | 0.0002 |
| Minocycline | D008911 | 1.88735456 | 0.028794 | 2.1372 | 0.014797 |
| monomethyl phthalate | C517284 | 1.9502095 | 0.028194 | 5.539215 | 0.0002 |
| monomethylarsonous acid | C406082 | 1.73652162 | 0.040992 | 4.424173 | 0.0002 |
| motexafin gadolinium | C437683 | 2.5496019 | 0.004599 | 3.416432 | 0.0006 |
| nickel monoxide | C028007 | 2.02873032 | 0.022196 | 5.693936 | 0.0002 |
| Omeprazole | D009853 | 2.49303214 | 0.006999 | 3.509456 | 0.0008 |
| Oxaliplatin | D000077150 | 2.28101611 | 0.011798 | 13.80277 | 0.0002 |
| Pentoxifylline | D010431 | 1.95340436 | 0.023795 | 2.590495 | 0.005199 |
| poly(propyleneimine) | C443641 | 1.82590892 | 0.035593 | 2.23635 | 0.011598 |
| polydatin | C058229 | 3.06605422 | 0.001 | 2.006423 | 0.019596 |
| Polyphenols | D059808 | 1.85712608 | 0.034593 | 2.171736 | 0.013397 |
| quinocetone | C502851 | 1.80767827 | 0.036393 | 3.928505 | 0.0002 |
| Raloxifene Hydrochloride | D020849 | 2.60716724 | 0.006599 | 6.147824 | 0.0002 |
| rottlerin | C085746 | 1.77438808 | 0.041392 | 2.834668 | 0.0022 |
| salinomycin | C010327 | 3.2518013 | 0.0008 | 3.229506 | 0.0012 |
| Selenium | D012643 | 1.87436961 | 0.031394 | 6.474139 | 0.0002 |
| selenomethylselenocysteine | C002979 | 2.94591252 | 0.001 | 1.749224 | 0.038192 |
| Smoke | D012906 | 1.76808816 | 0.040792 | 4.359221 | 0.0002 |
| sulindac sulfide | C025462 | 1.75091898 | 0.037393 | 4.056161 | 0.0002 |
| Surface-Active Agents | D013501 | 1.79095927 | 0.037992 | 1.661743 | 0.045991 |
| Tamoxifen | D013629 | 1.76289235 | 0.036593 | 10.30168 | 0.0002 |
| Temozolomide | D000077204 | 1.68407921 | 0.04919 | 2.062395 | 0.020596 |
| Tetrachloroethylene | D013750 | 1.7047072 | 0.047391 | 5.078841 | 0.0002 |
| thymoquinone | C003466 | 1.92697307 | 0.026995 | 3.199839 | 0.0006 |
| titanium dioxide | C009495 | 2.90992125 | 0.002599 | 10.40443 | 0.0002 |
| Topotecan | D019772 | 2.05998774 | 0.020996 | 14.27176 | 0.0002 |
| Trientine | D014266 | 1.66811257 | 0.046391 | 1.820386 | 0.031394 |
| U 0126 | C113580 | 2.25246112 | 0.012797 | 3.201393 | 0.001 |
| ursolic acid | C005466 | 1.84862336 | 0.034993 | 2.347875 | 0.009598 |
| Vigabatrin | D020888 | 2.02658999 | 0.022795 | 1.935292 | 0.025395 |
| Zinc Acetate | D019345 | 2.95392756 | 0.001 | 3.772735 | 0.0004 |
| 2-chloroethyl ethyl sulfide | C031278 | 1.82536887 | 0.029394 | 3.129137 | 0.0008 |
| Arsenic | D001151 | 1.71231665 | 0.046991 | 7.771464 | 0.0002 |
| Calcium | D002118 | 1.7389352 | 0.044191 | 2.095936 | 0.017596 |
| Calcium Chloride | D002122 | 1.76139283 | 0.036993 | 1.777146 | 0.035193 |
| CD 437 | C099555 | 1.75415222 | 0.036593 | 3.268486 | 0.0006 |
| Cholic Acids | D002793 | 1.80917311 | 0.031994 | 2.863243 | 0.0014 |
| Coal Ash | D060729 | 2.29396735 | 0.009398 | 2.164683 | 0.017397 |
| Dexrazoxane | D064730 | 1.99565224 | 0.021796 | 2.198421 | 0.012997 |
| Dextran Sulfate | D016264 | 1.98768723 | 0.026795 | 1.980763 | 0.025995 |
| Fenretinide | D017313 | 2.0510992 | 0.023195 | 12.68078 | 0.0002 |
| Levofloxacin | D064704 | 1.97544172 | 0.024795 | 2.505273 | 0.006199 |
| Mevalonic Acid | D008798 | 1.95960046 | 0.023595 | 2.784697 | 0.003399 |
| Mycophenolic Acid | D009173 | 2.10080178 | 0.016197 | 2.930743 | 0.0018 |
| PD 0325901 | C506614 | 1.74221921 | 0.034793 | 2.861421 | 0.0012 |
| Rotenone | D012402 | 1.95481058 | 0.026795 | 6.756841 | 0.0002 |
| RTKI cpd | C101044 | 1.78128838 | 0.033593 | 1.980941 | 0.026595 |
| Soman | D012999 | 2.37065967 | 0.009398 | 7.244134 | 0.0002 |
| Sterigmatocystin | D013241 | 1.68977171 | 0.042791 | 2.892111 | 0.002599 |
| testosterone-3-carboxymethyloxime-bovine serum albumin conjugate | C045037 | 1.6332029 | 0.04819 | 3.583505 | 0.0002 |
| tris(2-butoxyethyl) phosphate | C013320 | 1.71197388 | 0.047191 | 3.899963 | 0.0002 |
| Vitallium | D014800 | 2.4794375 | 0.005199 | 5.3198 | 0.0002 |

^1^ Chemical ID: The ID of the chemicals in [Comparative Toxicogenomics Database](http://ctdbase.org/)(CTD).

^2^P-value1: P value in GWAS dataset.

^3^NES1: Normalized enrichment score of GWAS dataset.

^4^P-value2: P value in mRNA expression profile.

^5^NES2: Normalized enrichment score of mRNA expression profile.

## Supplementary Table 5. 51 common chemicals identified for rectal cancer after a comparative of GWAS and mRNA GSEA results. (P value＜0.05)

| Chemical Name | Chemical ID^1^ | NES1^2^ | P-value1^3^ | NES2^4^ | P-value2^5^ |
| --- | --- | --- | --- | --- | --- |
| Polyethyleneimine | D011094 | 2.650559978 | 0.0029994 | 2.197126114 | 0.01159768 |
| Mitotane | D008939 | 2.752243607 | 0.00339932 | 1.884451824 | 0.030593881 |
| etomoxir | C054207 | 2.439751753 | 0.00379924 | 1.908468384 | 0.023195361 |
| sulindac sulfide | C025462 | 2.553718045 | 0.00519896 | 4.056160794 | 0.00019996 |
| Chlorambucil | D002699 | 2.522925084 | 0.00639872 | 2.125074539 | 0.012997401 |
| Brefeldin A | D020126 | 2.392726803 | 0.00859828 | 1.994192043 | 0.019996001 |
| Thiostrepton | D013883 | 2.407745203 | 0.00879824 | 2.782781514 | 0.00139972 |
| Amsacrine | D000677 | 2.220539425 | 0.01079784 | 2.637095236 | 0.0039992 |
| Methylnitronitrosoguanidine | D008769 | 2.289874973 | 0.013197361 | 3.735167752 | 0.00019996 |
| afimoxifene | C016601 | 2.296924572 | 0.013397321 | 5.957793363 | 0.00019996 |
| Tocopherols | D024505 | 2.085110195 | 0.016996601 | 2.319121867 | 0.00759848 |
| Nickel | D009532 | 2.105072089 | 0.017796441 | 12.37649043 | 0.00019996 |
| ochratoxin A | C025589 | 2.091291655 | 0.017996401 | 5.454982719 | 0.00019996 |
| Linoleic Acid | D019787 | 2.117730656 | 0.018396321 | 2.239156127 | 0.01019796 |
| Orlistat | D000077403 | 2.131308074 | 0.019596081 | 1.776258143 | 0.034993001 |
| Promethazine | D011398 | 1.991797113 | 0.019596081 | 3.166633685 | 0.00059988 |
| Aldosterone | D000450 | 2.049059527 | 0.019796041 | 2.061006979 | 0.016796641 |
| methyleugenol | C005223 | 2.030664412 | 0.020195961 | 2.216982202 | 0.01239752 |
| Mercaptopurine | D015122 | 2.026405171 | 0.023995201 | 3.900074273 | 0.00019996 |
| Thapsigargin | D019284 | 1.932255625 | 0.024395121 | 9.608383628 | 0.00019996 |
| Isoniazid | D007538 | 1.928859829 | 0.026194761 | 2.40781191 | 0.00679864 |
| incobotulinumtoxinA | C545476 | 1.969276639 | 0.027594481 | 9.911886354 | 0.00019996 |
| Cycloheximide | D003513 | 1.917881365 | 0.030993801 | 2.907226662 | 0.00179964 |
| Amino Acids | D000596 | 1.850353766 | 0.031193761 | 2.053157193 | 0.018396321 |
| usnic acid | C073339 | 1.808840109 | 0.034393121 | 3.134492009 | 0.00059988 |
| NADP | D009249 | 1.817930344 | 0.035792841 | 2.415426891 | 0.00779844 |
| LL-202 | C000589248 | 1.764501444 | 0.037392521 | 1.838122983 | 0.027794441 |
| Mitomycin | D016685 | 1.776706783 | 0.039592082 | 5.731968188 | 0.00019996 |
| lemongrass oil | C052901 | 1.775311621 | 0.039992002 | 4.008718941 | 0.00039992 |
| CC-8490 | C495817 | 1.715755252 | 0.040991802 | 2.44623928 | 0.0059988 |
| danthron | C004315 | 1.710871545 | 0.041791642 | 2.357388413 | 0.0089982 |
| Niacinamide | D009536 | 1.712740163 | 0.044191162 | 2.887496877 | 0.00179964 |
| importazole | C568452 | 1.681119537 | 0.044991002 | 1.899256238 | 0.022395521 |
| Quinic Acid | D011801 | 1.700698355 | 0.046190762 | 1.693080707 | 0.043591282 |
| pantogab | C016030 | 1.689387323 | 0.046390722 | 3.703391281 | 0.00039992 |
| Benomyl | D001542 | 1.647459945 | 0.047590482 | 2.180835274 | 0.012997401 |
| nodularin | C063998 | 1.666501886 | 0.048190362 | 2.147670992 | 0.012797441 |
| St. Thomas' Hospital cardioplegic solution | C041711 | 1.667150632 | 0.048590282 | 3.263338988 | 0.00079984 |
| Azoxymethane | D001397 | 1.623930473 | 0.049390122 | 3.118737582 | 0.00059988 |
| fumonisin B1 | C056933 | 2.971683522 | 0.00139972 | 2.726233216 | 0.00319936 |
| NAD | D009243 | 2.684430487 | 0.0019996 | 3.517521467 | 0.00039992 |
| casticin | C054133 | 2.231423929 | 0.00859828 | 3.725559824 | 0.00019996 |
| benz(a)anthracene | C030935 | 2.264296922 | 0.01239752 | 4.125719715 | 0.00019996 |
| 4-phenylbutyric acid | C075773 | 2.095061861 | 0.014997001 | 3.361958685 | 0.00039992 |
| beta Carotene | D019207 | 1.99633798 | 0.022595481 | 2.589517488 | 0.00379924 |
| Luteolin | D047311 | 1.878579225 | 0.027994401 | 2.5171115 | 0.00479904 |
| Plant Oils | D010938 | 1.848007895 | 0.032793441 | 2.133319434 | 0.016396721 |
| Colforsin | D005576 | 1.770014285 | 0.037592482 | 1.958830336 | 0.025194961 |
| Vancomycin | D014640 | 1.816854986 | 0.039192162 | 3.699502246 | 0.00019996 |
| PD 0325901 | C506614 | 1.68753574 | 0.040591882 | 2.861421046 | 0.00119976 |
| Toxaphene | D014112 | 1.643732972 | 0.049390122 | 2.013107999 | 0.021995601 |

^1^Chemical ID: The ID of the chemicals in [Comparative Toxicogenomics Database](http://ctdbase.org/)(CTD).

^2^P-value1: P value in GWAS dataset.

^3^NES1: Normalized enrichment score of GWAS dataset.

^4^P-value2: P value in mRNA expression profile.

^5^NES2: Normalized enrichment score of mRNA expression profile.
